# An Unexpected Encounter: Respiratory Syncytial Virus Nonstructural Protein 1 Interacts with Mediator Subunit MED25

**DOI:** 10.1128/jvi.01297-22

**Published:** 2022-09-14

**Authors:** Tessa Van Royen, Koen Sedeyn, George D. Moschonas, Wendy Toussaint, Marnik Vuylsteke, Delphi Van Haver, Francis Impens, Sven Eyckerman, Irma Lemmens, Jan Tavernier, Bert Schepens, Xavier Saelens

**Affiliations:** a VIB-UGent Center for Medical Biotechnology, VIB, Ghent, Belgium; b Department of Biochemistry and Microbiology, Ghent University, Ghent, Belgium; c VIB-UGent Center for Inflammation Research, VIB, Ghent, Belgium; d Department of Internal Medicine and Pediatrics, Ghent University, Ghent, Belgium; e Gnomixx, Melle, Belgium; f VIB Proteomics Core, Ghent, Belgium; g Department of Biomolecular Medicine, Ghent University, Ghent, Belgium; University of Kentucky College of Medicine

**Keywords:** MED25, interferon, nonstructural protein, protein-protein interactions, respiratory syncytial virus

## Abstract

Human respiratory syncytial virus (RSV) is the leading cause of severe acute lower respiratory tract infections in infants worldwide. Nonstructural protein NS1 of RSV modulates the host innate immune response by acting as an antagonist of type I and type III interferon (IFN) production and signaling in multiple ways. Likely, NS1 performs this function by interacting with different host proteins. In order to obtain a comprehensive overview of the NS1 interaction partners, we performed three complementary protein-protein interaction screens, i.e., BioID, MAPPIT, and KISS. To closely mimic a natural infection, the BioID proximity screen was performed using a recombinant RSV in which the NS1 protein is fused to a biotin ligase. Remarkably, MED25, a subunit of the Mediator complex, was identified in all three performed screening methods as a potential NS1-interacting protein. We confirmed the interaction between MED25 and RSV NS1 by coimmunoprecipitation, not only upon overexpression of NS1 but also with endogenous NS1 during RSV infection. We also demonstrate that the replication of RSV can be enhanced in MED25 knockout A549 cells, suggesting a potential antiviral role of MED25 during RSV infection. Mediator subunits function as transcriptional coactivators and are involved in transcriptional regulation of their target genes. Therefore, the interaction between RSV NS1 and cellular MED25 might be beneficial for RSV during infection by affecting host transcription and the host immune response to infection.

**IMPORTANCE** Innate immune responses, including the production of type I and III interferons, play a crucial role in the first line of defense against RSV infection. However, only a poor induction of type I IFNs is observed during RSV infection, suggesting that RSV has evolved mechanisms to prevent type I IFN expression by the infected host cell. A unique RSV protein, NS1, is largely responsible for this effect, probably through interaction with multiple host proteins. A better understanding of the interactions that occur between RSV NS1 and host proteins may help to identify targets for an effective antiviral therapy. We addressed this question by performing three complementary protein-protein interaction screens and identified MED25 as an RSV NS1-interacting protein. We propose a role in innate anti-RSV defense for this Mediator complex subunit.

## INTRODUCTION

Human respiratory syncytial virus (RSV) is an enveloped, negative-stranded RNA virus that is classified in the *Pneumoviridae* family of the order *Mononegavirales*. RSV is a common cause of acute lower respiratory tract infections that require medical attention in infants, the elderly, and the immunocompromised (1, 2). In children below 5 years of age, it is estimated that approximately 84,500 to 125,200 deaths are caused annually by RSV-associated acute lower respiratory tract infections, with most of these fatalities occurring in low- and middle-income countries (3). Annually, RSV is estimated to be responsible for 14,000 in-hospital deaths in older adults worldwide (4). Despite the global disease burden of RSV, no approved vaccine or effective treatment is available. A better understanding of the interactions that occur between RSV and host proteins may help to identify targets for an effective antiviral therapy.

The RSV genome is composed of approximately 15,200 nucleotides and codes for 11 proteins. RSV can be distinguished from most other viruses of the *Mononegavirales* by the presence of two nonstructural proteins, NS1 and NS2 (5). Due to the 3′-proximal location on the viral genome, the NS genes are the most abundantly transcribed viral genes. NS1 and NS2 are known to strongly reduce the induction and signaling of type I and type III interferon (IFN) during RSV infection by acting individually and cooperatively in multiple ways. NS1 (139 amino acid residues) and NS2 (124 amino acid residues) are relatively small proteins, and it is suggested that they interact with host proteins to exert their effector functions. NS1, for example, interacts with mitochondrial antiviral signaling protein (MAVS) and thereby prevents the interaction between MAVS and retinoic acid-inducible gene I (RIG-I), resulting in a blunted RIG-I-like receptor-mediated IFN induction (6). In addition, NS1 has been shown to interact with the ubiquitin ligase TRIM25, which is crucial for RIG-I activation (7). NS2 can directly bind to RIG-I, thereby disrupting the interaction of RIG-I with MAVS (8). Downstream of MAVS, both NS1 and NS2 can interfere with the activation of IFN regulatory factors 3 and 7 (IRF3 and IRF7), which disturbs their effector functions (9–11). Signal transducer and activator of transcription 2 (STAT2) protein levels are reduced upon RSV infection, but to date, it is still debated whether mainly NS1, NS2, or both are responsible for this reduction (12–15). It is hypothesized that, overall, NS1 is the major IFN antagonist and that this antagonism is enhanced in the presence of NS2 (16, 17). Crystal structure analysis has revealed that NS1 is built up as a β-sandwich flanked by three α-helices (18). This fold of NS1 is similar to the N-terminal domain of RSV M, but, in addition, NS1 has a unique α-helix at its C terminus. This helix, named α3 (amino acid residues 119 to 139), is critical for the inhibition of IFN expression, as truncation of this helix or mutation of three residues (Y125, L132, and L133) results in decreased NS1-mediated suppression of IFN-β induction (18).

Until recently, only the cytoplasmic effect of NS1 in the control of IFN responses was well described. In the absence of NS2, NS1 expression after transient transfection is detected in both the cytoplasm and the nucleus (10, 11, 19–21). However, coexpression of NS2 seems to induce mitochondrial localization of NS1, which appears to be dependent on MAVS (13, 19). Interestingly, Pei et al. reported that NS1 also partitions into the nucleus during an RSV infection, which helps to explain its multifunctional nature (22). A proteomics screen to investigate RSV NS1-interacting proteins has been performed by overexpression of a green fluorescent protein (GFP)-tagged NS1, which revealed 221 candidate NS1-interacting proteins (23). Some of these candidate interactors are known to be involved in the regulation of the cell cycle.

To better understand the diverse effector functions of NS1, we aimed to identify new host proteins that interact with NS1. For this, we performed three complementary protein-protein interaction mapping screens, i.e., BioID, MAPPIT, and KISS. Since each protein interaction requires specific conditions to be detected, we reasoned that combining complementary techniques might offer a unique insight into the NS1 interactome (24). To our knowledge, these techniques have not yet been applied to identify candidate RSV NS1 interactors.

BioID is based on the fusion of a highly promiscuous mutant of the Escherichia coli BirA biotin ligase (designated BirA*) to a bait protein (25). This way, BioID allows sampling of the bait proxeome in the intact cell by enriching biotinylated proteins that are subsequently identified by mass spectrometry. Since the biotin labeling step occurs before cell lysis, BioID has the advantages that bait-interacting proteins are labeled in their natural cellular context and that both weak and transient interactions can be detected. In this study, we aimed to perform a BioID proximity screen in the context of an RSV infection. Therefore, we generated a recombinant RSV in which we fused BirA* to the C terminus of NS1. As such, proteins that are in the close proximity to NS1 during infection can be biotinylated and identified by mass spectrometry. To our knowledge, this is the first time that NS1 host-interacting proteins are investigated during RSV infection.

We also set out to investigate the NS1 interactome with two binary methods, MAPPIT and KISS, which are based on cytokine signal transduction (26, 27). Briefly, MAPPIT uses an engineered chimeric receptor that consists of the extracellular part of the erythropoietin (EPO) and the cytosolic part of the leptin receptor (28). Three tyrosine residues in the cytosolic part of this chimeric receptor are mutated to abolish downstream signaling through STAT3 recruitment upon binding of EPO, the ligand. A bait protein is fused to the cytosolic portion of this chimeric receptor, and a library of prey proteins is fused to the C-terminal part of the gp130 chain that carries four functional STAT3 recruitment sites. In the presence of EPO and upon bait and prey interaction, these STAT3 recruitment sites are activated, leading to downstream signaling and activation of a STAT-responsive reporter gene. KISS is based on the same principle as MAPPIT except that in KISS, the bait protein is fused to the kinase domain of TYK2, a cytosolic protein (29). For both MAPPIT and KISS, the simple reporter gene readout enables high-throughput screening for covalent, transient, and indirect interactions in intact mammalian cells. It is important to note that in MAPPIT, the bait is anchored to the plasma membrane, which may not be the native subcellular localization of the bait, whereas in KISS, the bait is expressed as a cytosolic fusion protein (27).

In this study, we identified RSV NS1-interacting proteins with three complementary screening methods and focused on MED25, a subunit of the Mediator complex, as one of the interaction partners that was identified in the three screens.

## RESULTS

### Generation and characterization of recombinant RSVs that express NS1-BirA* or mKate2-BirA*.

**FIG 1:**
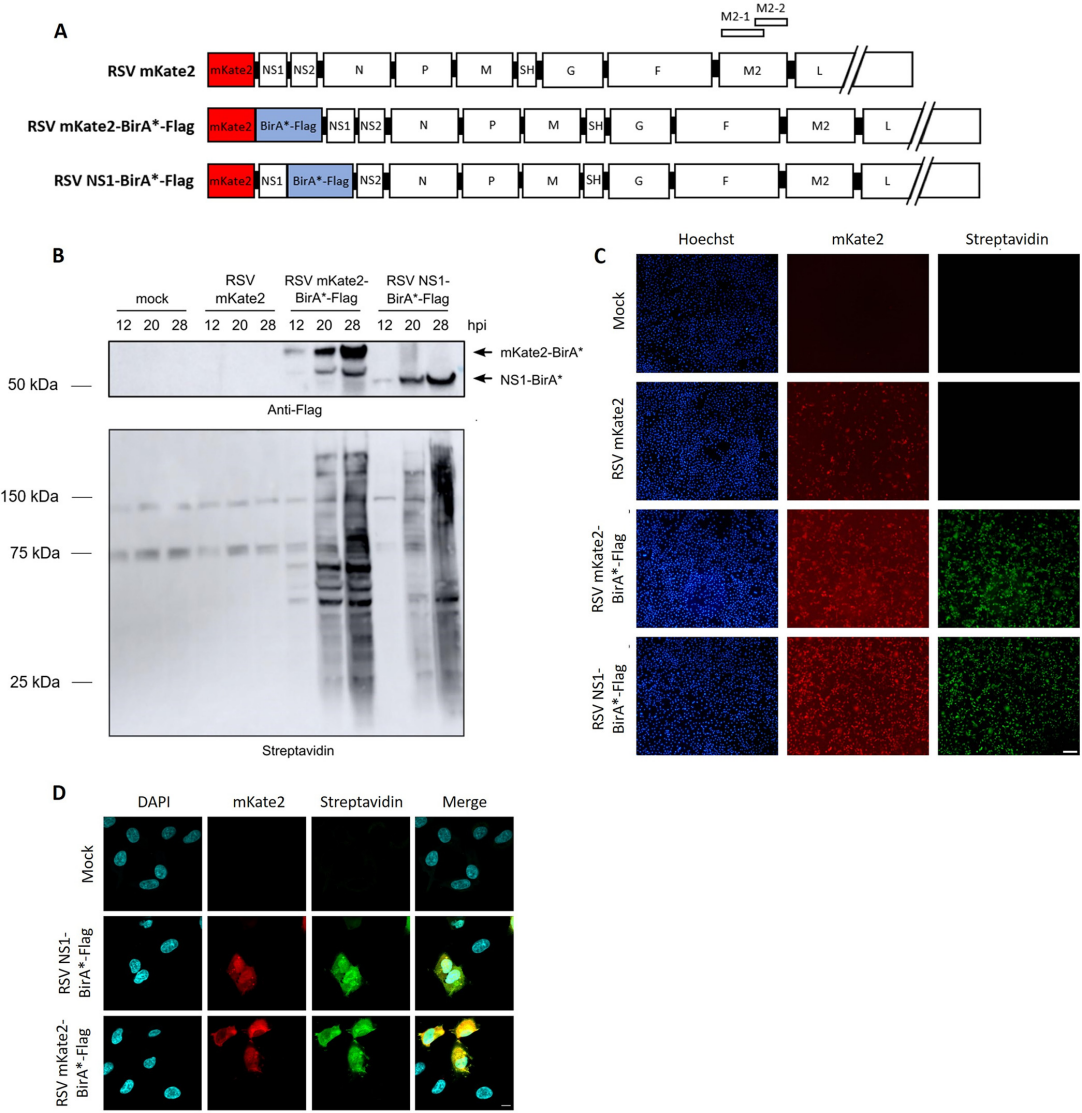
Characterization of recombinant RSVs expressing NS1-BirA* or mKate2-BirA*. (A) Genome organization (antigenomic cDNA) of recombinant parental RSV mKate2 and RSV expressing NS1-BirA* or mKate2-BirA*. (B) Immunoblot showing expression of NS1-BirA*-Flag and mKate2-BirA*-Flag fusion proteins (upper panel) and protein biotinylation upon expression of the fusion proteins (lower panel). A549 cells were infected with the recombinant BirA*-expressing RSVs (MOI, 1) or the control RSV mKate2 or were mock infected for 4 h, followed by incubation of the cells with 50 μM biotin for an additional 8, 16, or 24 h. Cell lysates were analyzed by Western blotting with an anti-Flag antibody or streptavidin. (C) Immunofluorescence images after infection of A549 cells with RSV NS1-BirA* and mKate2-BirA* (MOI, 1) for 4 h, followed by incubation with biotin (50 μM) for an additional 16 h. Cells were fixed and stained with streptavidin-AF488 and Hoechst stain. Scale bar, 0.1 mm. (D) A549 cells were infected with the indicated recombinant BirA*-expressing RSVs at an MOI of 1 or were mock infected for 4 h, followed by incubation of the cells with 50 μM biotin for an additional 16 h. The cells were then fixed and stained with streptavidin-AF488 and DAPI. The cells were imaged by confocal immunofluorescence microscopy. Scale bar, 10 μm.

To identify host proteins that interact with NS1 in the course of an RSV infection, we performed a BioID screen in the human lung carcinoma cell line A549, which is permissive for RSV. Such a proximity labeling screen has not yet been reported in the context of an RSV infection. Therefore, we generated a recombinant RSV Katushka 2 (mKate2) reporter virus in which Flag-tagged BirA* was genetically fused to the C terminus of NS1. This virus, derived from RSV A2-line19F, was generated with a bacterial artificial chromosome (BAC) and was named RSV NS1-BirA*-Flag (30, 31). We used the BAC plasmid pSynkRSV-line19F that contains the antigenomic cDNA of RSV genotype A2-line19F with an mKate2 fluorescence reporter gene. In addition, as a control for the BioID screen, we also rescued RSV mKate2-BirA*-Flag in which the Flag-tagged BirA* gene was fused to the C terminus of mKate2. The genome organizations of the rescued BirA*-encoding viruses and the parental RSV mKate2 virus are represented in Fig. 1A. The three recombinant viruses were subjected to metagenomics sequencing, revealing that the NS1- and mKate2-BirA* sequences were not altered in comparison to the corresponding BAC vectors that were used to rescue the viruses.

We first investigated the biotinylation activity of these viruses as well as the expression of the created BirA* fusion constructs by Western blotting. A549 cells were infected with different multiplicities of infection (MOIs) of the parental RSV mKate2, RSV mKate2-BirA*-Flag, or RSV NS1-BirA*-Flag for 4 h, followed by incubation of the cells with 50 μM biotin for an additional 8, 16, or 24 h. Expression of NS1- and mKate2-BirA* fusion proteins was detectable starting from 12 h postinfection, when the cells were infected at an MOI of 1 or 2.5 (Fig. 1B and see Fig. S1 in the supplemental material). Expression of these proteins increased over time. Protein biotinylation was observed at 20 and 28 h after infection with both RSV mKate2-BirA-Flag* and NS1-BirA*-Flag (Fig. 1B). In mock-infected or RSV mKate2-infected cells, only three streptavidin-reactive bands were observed. These bands likely represent naturally biotinylated proteins, corresponding to carboxylase proteins in mammalian cells (32).

Subsequently, we investigated the infectivity of and *in situ* protein biotinylation activity by the recombinant RSVs by monitoring mKate2 expression and streptavidin binding by immunofluorescence microscopy. Infection of A549 cells with RSV NS1-BirA*-Flag or RSV mKate2-BirA*-Flag (MOI, 1) resulted in a comparable number of mKate2-positive cells and streptavidin reactivity (Fig. 1C). Finally, we analyzed the biotinylation activity of RSV NS1-BirA*-Flag and RSV mKate2-BirA*-Flag in infected A549 cells by confocal microscopy, to picture the subcellular localization of the BirA* fusion proteins (33). In both RSV NS1-BirA*-Flag-infected cells and RSV mKate2-BirA*-Flag-infected cells, we observed streptavidin reactivity in the cytoplasm as well as in the nucleus (Fig. 1D). The streptavidin staining seemed more pronounced in the nuclei of RSV NS1-BirA*-Flag-infected cells than in the nuclei RSV mKate2-BirA*-Flag-infected cells. Overall, these results show that the rescued RSV mKate2-BirA*-Flag and RSV NS1-BirA*-Flag viruses express the respective BirA*-Flag fusion proteins, which are enzymatically active.

### BioID screen with recombinant RSV NS1-BirA*-Flag reveals enrichment of Mediator complex subunits.

**FIG 2:**
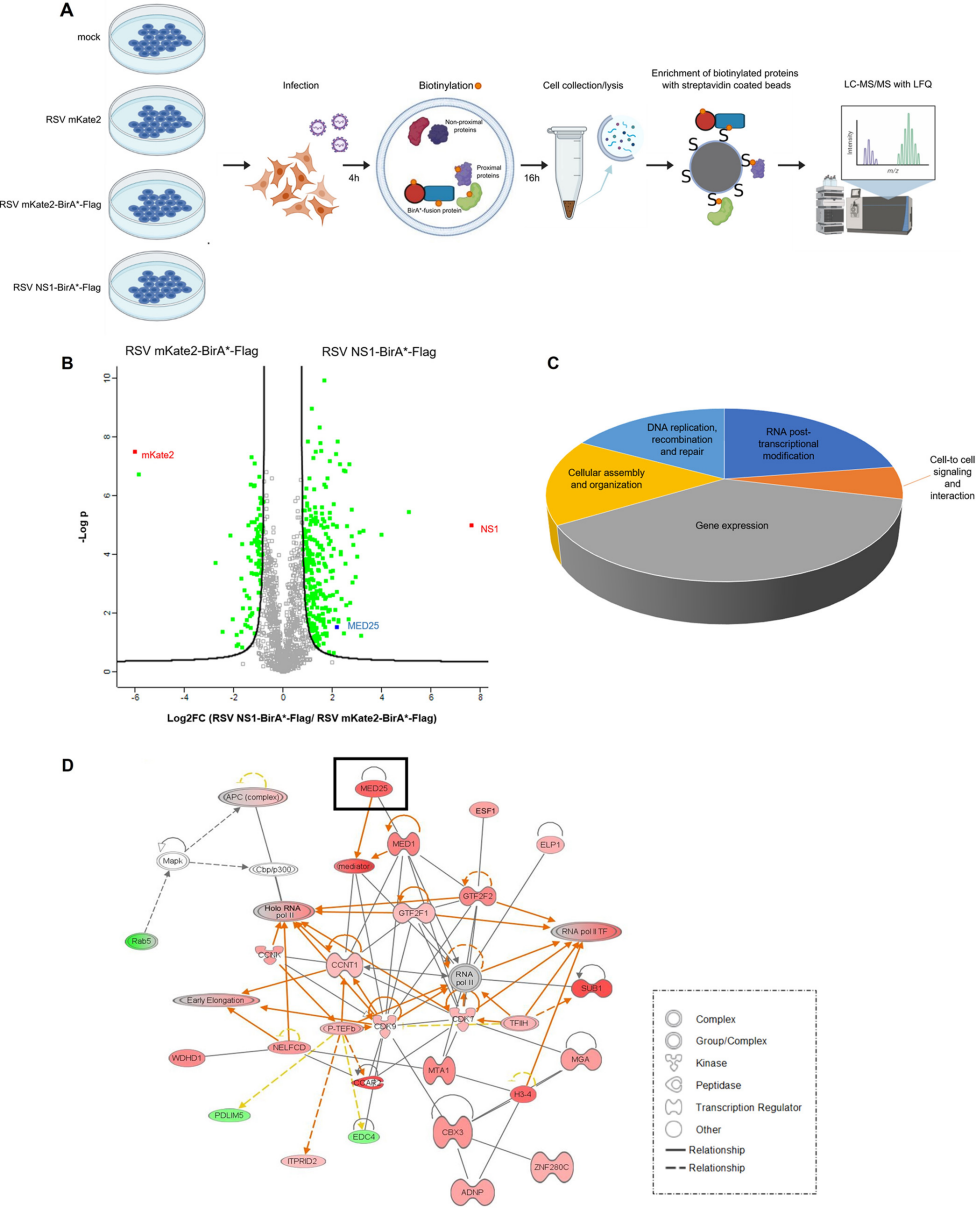
BioID screen with recombinant NS1-BirA*- and mKate2-BirA*-expressing RSV. (A) Schematic representation of the setup of the BioID screen with recombinant RSV NS1-BirA*-Flag and RSV mKate2-BirA*-Flag. A549 cells were infected (MOI, 1) for 4 h, followed by the addition of biotin (50 μM) for 16 h to biotinylate proteins in the proximity of NS1 and mKate2, respectively. After cell lysis (performed at 20 hpi), the biotinylated proteins were enriched on streptavidin-conjugated beads. The streptavidin-purified proteins were digested by trypsin on the beads, and the peptides were identified and quantified by LC-MS/MS analysis. The figure was created with BioRender software. (B) Volcano plot demonstrating fold change enrichment of host proteins in NS1-BirA* RSV-infected cells compared to mKate2-BirA* RSV-infected cells. Significantly enriched candidate interactors are depicted in green, blue (MED25), or red (NS1 and mKate2), and other identified proteins are depicted in gray (not significant). (C) Bioinformatics analysis of proteins that were identified in the BioID screen. All identified proteins were uploaded in the IPA software, with a filtering for significantly enriched proteins as indicated in panel B. An enrichment analysis was performed by IPA to assign molecular and cellular functions to the proteins enriched in the RSV NS1-BirA*-Flag-infected cells. (D) IPA software was used to explore the connections between proteins identified in the BioID screen in RSV NS1-BirA*-Flag-infected cells. Red and green nodes indicate proteins that are, respectively, significantly increased and decreased in abundance in RSV NS1-BirA*-Flag-infected cells compared to RSV mKate2-BirA*-Flag-infected cells, and the color intensity corresponds to the degree of abundance. White nodes represent proteins identified through the Ingenuity Pathways Knowledge Base. The biological relationship between two nodes is represented by a line. Solid and dashed lines indicate direct and indirect molecular relationships, respectively.

We next performed a BioID screen with RSV NS1-BirA*-Flag and RSV mKate2-BirA*-Flag as a control to identify the RSV NS1 proxeome (Fig. 2A). Four biological replicates of A549 cells were infected (MOI, 1) with RSV NS1-BirA*-Flag or RSV mKate2-BirA*-Flag for 4 h, followed by the addition of biotin (50 μM). Twenty hours after infection, a time point at which NS1-BirA*-Flag- or mKate2-BirA*-Flag-mediated biotinylation of proteins in the proximity of NS1 or mKate2, respectively, is ongoing (Fig. 1B), the infected cells were lysed. In addition, two biological replicates of A549 cells that were mock infected or infected with parental RSV mKate2 were used as negative controls. After cell lysis, the biotinylated proteins that represent candidate interactors of NS1 and mKate2 were enriched on streptavidin-conjugated beads. The streptavidin-binding proteins were digested with trypsin on the beads, and the peptides were further identified and quantified by liquid chromatography-tandem mass spectrometry (LC-MS/MS) (Fig. 2A). In total, we could quantify 1,400 proteins that were present in at least three out of four biological replicates after infection with RSV NS1-BirA*-Flag and/or RSV mKate2-BirA*-Flag. To distinguish proximity partners specific for NS1, for each quantified protein, the intensities in the RSV NS1-BirA*-Flag-infected and RSV mKate2-BirA*-Flag-infected samples were compared by *t* testing. As such, we could detect 271 host proxeome partners of RSV NS1 (Fig. 2B and Table S1). Inversely, 70 host proteins were significantly enriched in the RSV mKate2-BirA*-Flag-infected cells. Of the 341 significantly enriched proteins that are highlighted in the volcano plot, we designed a heat map in which imputed values are removed (Fig. S2).

The biological relevance of the BioID data was then explored using the Ingenuity Pathway Analysis (IPA) software that allows assignment of functions and creation of networks in high-throughput lists of proteins by using bundled information from literature and from curated databases (34). A core analysis performed in IPA revealed that many proteins of the NS1 proxeome are involved in gene expression and posttranscriptional modification of RNA (Fig. 2C). Subsequently, a network analysis was performed in which the identified candidate NS1-interacting proteins were mapped against all networks included in the IPA software. One of the top-ranked connected networks revealed that RSV NS1 may interact with several subunits of the Mediator complex, including MED25 (Fig. 2D).

### MAPPIT and KISS screens reveal MED25 as a NS1 interaction partner.

**FIG 3:**
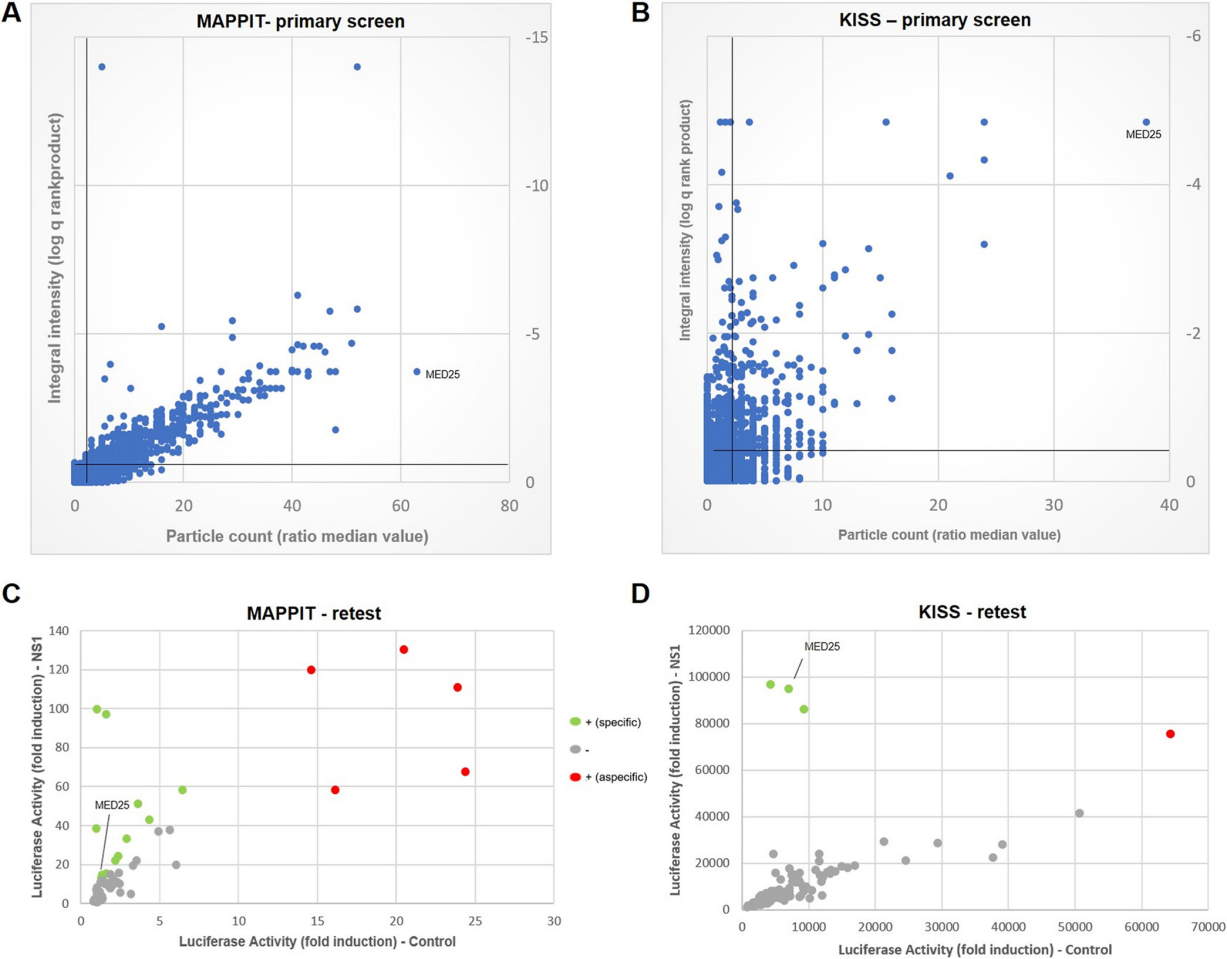
MAPPIT and KISS identify MED25 as a candidate NS1-interacting protein. (A) Results of the MAPPIT primary screening. A library covering 14,817 open reading frame (ORF) preys was screened with the NS1 bait chimera, with each ORF yielding quadruplicate samples for both the stimulated (+Epo) and unstimulated (−Epo) conditions. The plot represents the log-transformed *q* value of the ratio (stimulated versus unstimulated samples) of normalized MAPPIT luciferase activity versus the ratio (stimulated versus unstimulated samples) of the median value of fluorescence particle counts. (B) Results of the KISS primary screening. Each prey is transfected 4-fold with either the specific bait (NS1) or no bait (TYK2 only) as a control. The plot represents the log-transformed *q* value of the ratio (specific bait versus TYK2 only) of normalized KISS luciferase activity versus the ratio (specific bait versus TYK2 only) of the median value of fluorescence particle counts. For both panels A and B, a threshold *q* value of <0.35 and a particle count of >2 were applied, as these result in a high specificity (low false-positive rate). (C and D) Plots showing, respectively, the MAPPIT and KISS retests of 96 proteins of interest (list available in Table S4 in the supplemental material). The ORF preys were reevaluated by testing their interaction with either NS1 or a negative control bait. For MAPPIT, the fold induction of the average luciferase activity of triplicate EPO-stimulated versus unstimulated samples for either bait is shown. For KISS, the average luciferase activity for interaction with either bait is shown. A set of previously defined criteria for sensitive and specific MAPPIT and KISS analysis is applied (67): signals were scored negative (−; gray) or positive (+). Positive signals were further categorized as corresponding with an aspecific interaction (red) where the ORF prey binds to a component of the control bait rather than the NS1 bait or an NS1-specific interaction (green).

To complement the BioID screen, we performed two binary RSV A2 NS1 interaction screens using MAPPIT and KISS. As such, we wanted to obtain a more comprehensive overview of candidate NS1 interaction partners and intended to focus on overlapping hits. MAPPIT and KISS are two-hybrid approaches that are based on the complementation of cytokine signal transduction (26–29). The prey collection that was used in the MAPPIT and KISS screens consists of 14,817 proteins, selected from the human ORFeome collection version 8.1 and the ORFeome collaboration (OC) collection (35, 36). During an automated primary MAPPIT screening of this library with the NS1 bait chimera, transfected cells were either stimulated with EPO or left untreated. For the primary KISS screen, each prey of the library was transfected either with the NS1 bait or without bait as a control. After 24 h, interactions were monitored by measuring luciferase reporter activity (Fig. 3A and B). These primary screens revealed 380 and 475 potential NS1-interacting proteins for MAPPIT (Table S2) and KISS (Table S3), respectively. Of these, MED25 was again identified as an NS1 interaction partner with both screening methods.

After these primary screens of the full prey collection, we generated a selected protein pick list consisting of 96 preys (Table S4) that was reevaluated for interaction with the NS1 bait or with a negative control bait in a manual MAPPIT and KISS binary retest. This pick list included candidates that had been identified in the BioID screen as well as a number of reported NS1 interactors available in the prey collection, such as cullin-2, MAP1B, H2BD, and IRF3 (9, 19, 20). In addition, we included several key proteins that are involved in the induction and signaling of type I and type III IFNs upon RSV infection, such as STAT1, STAT2, TLR3, TBK1, MDA5, MyD88, TRIF, and IKKε (8, 12, 37–42). The results of this retest for MAPPIT and KISS are shown in Fig. 3C and D. Interestingly, three different host proteins tested positive for interaction with NS1 in both the MAPPIT and KISS retest: MSRA, NOL4, and MED25 (Table S4). To our knowledge, no relevant function has yet been described for MSRA or NOL4 during a viral infection. It has recently been reported that RSV NS1 coimmunoprecipitates with the Mediator complex and, in this way, may modulate expression of host genes, including antiviral genes (22). Interestingly, EP300, which is involved in gene transcription via chromatin remodeling, was also identified as an NS1 interactor in the MAPPIT retest (Table S4) (43).

### Candidate NS1 interactors identified in all three protein-protein interaction screens.

**FIG 4:**
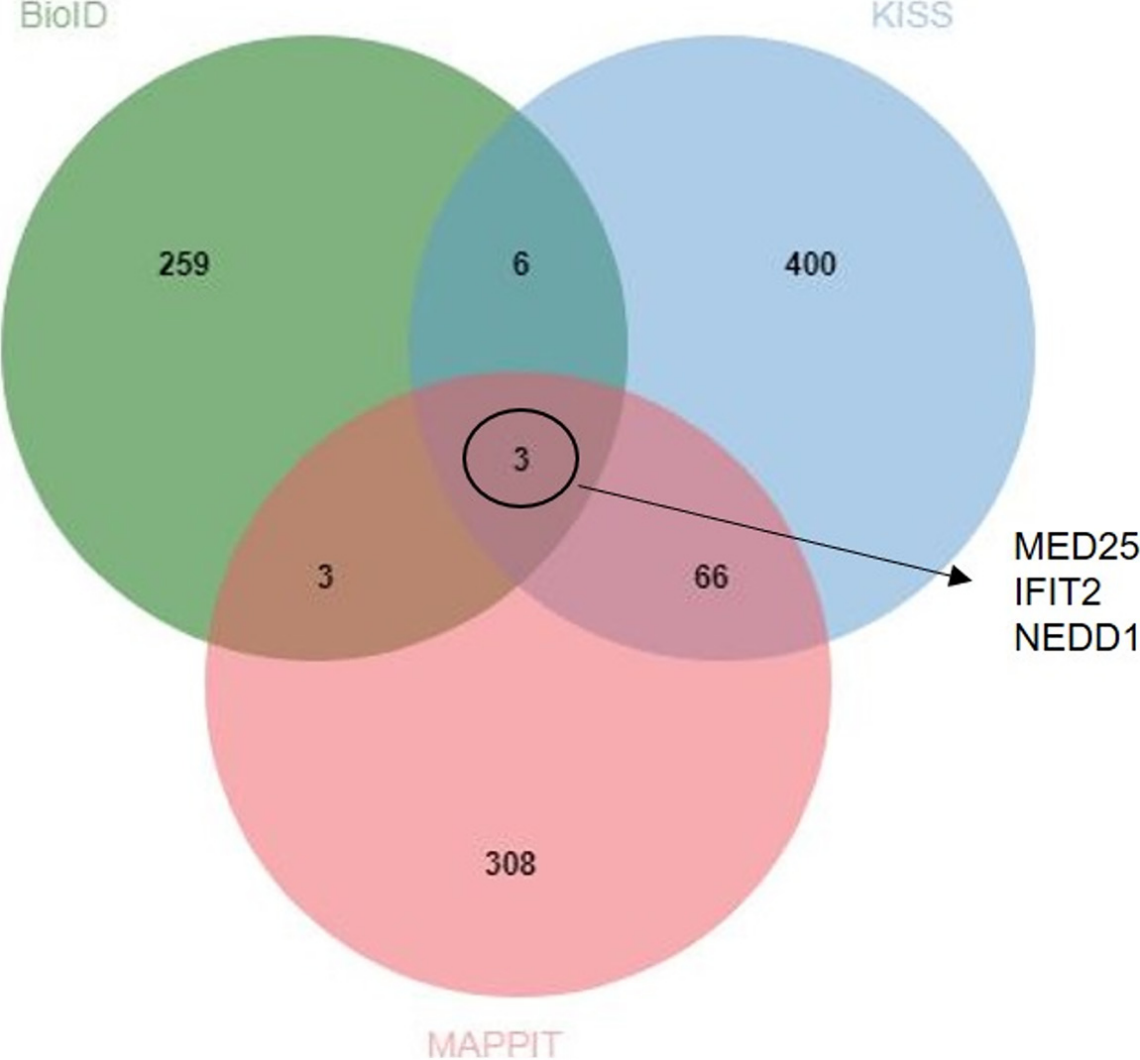
Venn diagram depicting the overlap of NS1 candidate interactors between the BioID, MAPPIT, and KISS screening methods. The BioID data set (significantly positively enriched proteins in RSV NS1-BirA*-Flag-infected samples relative to RSV mKate2-BirA*-Flag-infected samples), the MAPPIT and KISS data sets (filtered data set [*q* value of <0.35, particle counts of >2, removal of known aspecific binders] after primary screen against the 15,000 ORF prey collection) were imported into the jvenn online tool to identify proteins in common.

To further validate the candidate NS1 interactors, we looked for proteins in common that were identified in all three protein-protein interaction screens. Three proteins, MED25, interferon-induced protein with tetratricopeptide repeats 2 (IFIT2), and neural precursor cell expressed, developmentally downregulated 1 (NEDD1), were identified in all three screens (Fig. 4).

Furthermore, Table 1 provides an overview of NS1 candidate interactors that were shared by at least two of the three performed protein-protein interaction screens. The functions of these proteins in common are summarized in Table S5. Interestingly, several proteins with E3 ubiquitin ligase activity were identified (i.e., COMMD5, KCMF1, SH3RF2, ANKRD9, UHRF2, FBXO11, and DTX3L), suggesting that NS1 hijacks the host ubiquitin-proteasome system, which has been reported previously (12, 13).

**TABLE 1:** Candidate interactors of NS1 identified in at least two of the three protein-protein interaction mapping techniques (i.e., the BioID, MAPPIT, and KISS screens)

Screens	Proteins in common
BioID, MAPPIT, and KISS	NEDD1, MED25, IFIT2
MAPPIT and KISS	FRG1B, MSRA, GUSBP2, AZU1, TTC19, STAT5B, RAC1, ACAD11, NOL4, ADD3, PRPS2, ABRA, BRIX1, CLEC18C, COMMD5, SGOL1, ARMC12, PRKCA, CXorf21, DEPDC7, C16orf45, CCDC125, HSP90AB1, CTNNA3, KCMF1, CCDC65, TGFBR1, CENPP, KIAA1217, PDLIM7, EPB41L1, CAMK2A, RFC5, C10orf122, SPANXN3, PATL1, COPS2, WHAMMP3, SH3RF2, CEP89, GAST, CCDC158, VPS39, NLRP10, ANKRD9, UHRF2, MORC2-AS1, IGKC, HAL, CCDC114, SLC44A5, TREX1, KIAA1683, XRCC68P1, FBXO11, STBD1, THAP8, FAM98A, LOC254896, ERCC5, KRT6C, PRG3, GTF2E2, SULF2, ASIP
BioID and MAPPIT	CNOT10, DTX3L, CSTF2T
BioID and KISS	KLC1, KIF23, CCZ1B, TMEM199, VCPIP1, SQSTM1

### Validation of the NS1-MED25 interaction.

**FIG 5:**
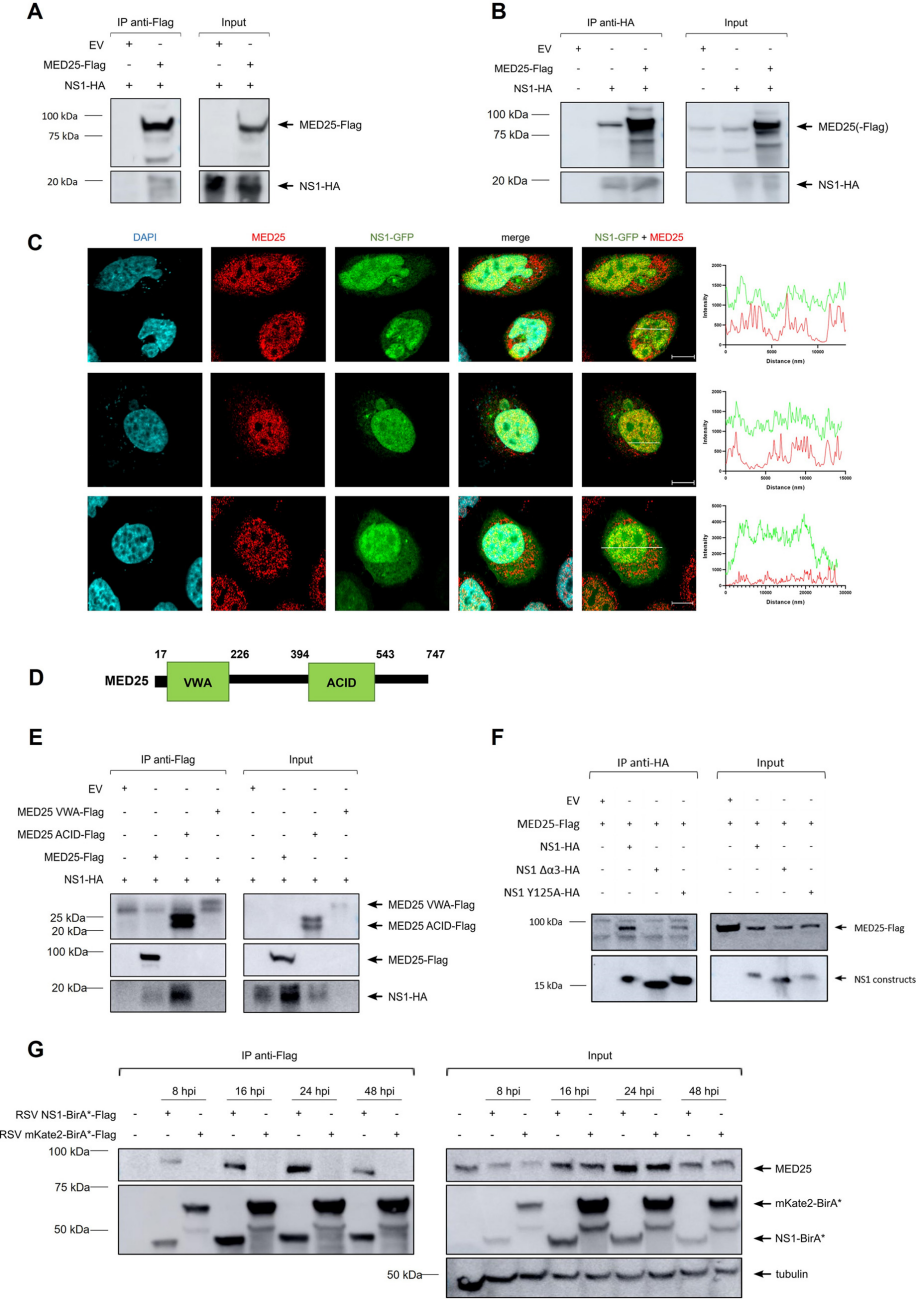
RSV NS1 binds to MED25. (A) Anti-Flag pulldown shows coimmunoprecipitation of NS1-HA with MED25-Flag but not with empty vector (EV) control after transient overexpression in HEK293T cells. Blots were analyzed with anti-Flag and anti-HA antibodies. (B) Anti-HA pulldown shows coimmunoprecipitation of MED25-Flag (left panel, lane 3) and endogenous MED25 (left panel, lane 2) with NS1-HA but not with EV control (left panel, lane 1). Blots were analyzed with anti-MED25 and anti-HA antibodies. (C) Representative confocal micrographs of HeLa cells that were transfected with NS1-GFP. Cells were fixed and stained with anti-MED25 antibody (red) or DAPI (blue). Scale bar, 10 μm. The three rows represent three different confocal images. The right panels show the intensity profile analysis along the white line (using ZEN 3.4 Blue software) to display the distribution of the red (MED25) and green (NS1-GFP) fluorophore signals. (D) Schematic representation of the MED25 domains. (E) Anti-Flag pulldown shows coimmunoprecipitation of NS1-HA with full-length MED25 (left panel, lane 2) and MED25 ACID (left panel, lane 3) but not with MED25 VWA (left panel, lane 4) or with EV (left panel, lane 1). Blots were analyzed with anti-Flag and anti-HA antibodies. (F) Anti-HA pulldown shows coimmunoprecipitation of MED25-Flag with NS1-HA (left panel, lane 2) but not with NS1 Δα3-HA (left panel, lane 3) or with EV (left panel, lane 1). MED25-Flag coimmunoprecipitates with NS1 Y125A-HA but less well than with NS1-HA. (G) A549 cells were infected for 8, 16, 24, or 48 h with NS1-BirA*- or mKate2-BirA*-expressing RSV at an MOI of 1, 0.5, 0.1, or 0.01, respectively. Anti-Flag pulldown shows coimmunoprecipitation of MED25 with NS1-BirA*-Flag for every virus incubation time but not with mKate2-BirA*-Flag (left panel). Blots were analyzed with anti-MED25, anti-Flag, and anti-tubulin antibodies.

After the identification of MED25 as a candidate interactor of NS1 in the three complementary screens, this interaction was confirmed by coimmunoprecipitation experiments using lysates of transfected HEK293T cells (Fig. 5A). In addition, endogenous MED25 could be coimmunoprecipitated with NS1 (Fig. 5B). We also analyzed the subcellular localization of NS1-GFP and endogenous MED25. In HeLa cells transfected with an NS1-GFP expression vector, the localization of NS1-GFP was primarily observed in the nucleus and, although less pronounced, also in the cytoplasm (Fig. 5C). The presence of overexpressed NS1 in both the nucleus and cytoplasm is in accordance with previous reports (19, 22). However, NS1-GFP was predominantly localized in the nucleus of the transfected HeLa cells as used here, whereas in those reports, in which transfected A549 cells were imaged, NS1(-GFP) is more clearly present in the cytoplasm. Immunostaining of cells with a MED25-specific antibody resulted in both nuclear and cytoplasmic reactivity, which differs from the expected nuclear localization of MED25 (Fig. 5C) (44). However, when MED25 knockout A549 cells (described below in “Replication of RSV B1 is significantly enhanced in MED25 knockout A540 cells”) were stained with this anti-MED25 antibody, a comparable cytoplasmic but not nuclear staining was observed (Fig. S3). These results suggest that the cytoplasmic staining of MED25 is likely due to aspecific binding of the MED25 antibody. Hence, these observations illustrate that MED25 and NS1-GFP both localize in the nucleus.

MED25 protein comprises two domains: an N-terminal von Willebrand factor type A domain (VWA) and a central activator interacting domain (ACID) (Fig. 5D). The MED25 VWA domain directs MED25 incorporation into the Mediator complex, while the MED25 ACID domain interacts with transcriptional activation domains (TADs) of different DNA-binding transcriptional activators. In addition to cellular transcription factors that bind to MED25 ACID, the herpes simplex virus transactivator protein VP16 also targets this domain of MED25 (45–49).

To provide further insight into the possible biological effect of the NS1-MED25 interaction, we investigated which domain of MED25 is targeted by NS1. For this, HEK293T cells were transfected with NS1-hemagglutinin (HA)-tagged and Flag-tagged MED25 expression constructs (VWA domain only, ACID domain only, or full-length MED25). Interestingly, the expression level of NS1-HA was reduced in the presence of MED25 ACID-Flag and even undetectable when MED25 VWA-Flag was cotransfected (Fig. 5E, right panel). The expression level of MED25 VWA-Flag itself was also reduced in the presence of NS1-Flag, whereas transfection of MED25 VWA-Flag alone in HEK293T cells resulted in clearly detectable expression of this domain (Fig. 5E and Fig. S4). NS1-HA coimmunoprecipitated with full-length MED25-Flag and with MED25 ACID-Flag (Fig. 5E). These results suggest that the ACID domain of MED25 is responsible for its interaction with NS1. However, a possible contribution of the MED25 VWA domain for the interaction with NS1 could not be excluded from these experiments because the candidate interaction partners were very poorly expressed upon cotransfection.

The C-terminal α3 helix of NS1 was reported to play a crucial role in the modulation of host responses, since the NS1 Y125A and NS1 Δα3 mutants partially lose their ability to suppress IFN induction and differentially affect host gene expression compared to wild-type NS1 (18, 22). Removal of the NS1 α3 helix abrogated the interaction between NS1 and MED25-Flag, whereas the Y125A mutation reduced the interaction (Fig. 5F). These findings are in accordance with the recent study by Dong et al. (50).

We next evaluated the interaction between NS1 and MED25 during an RSV infection in A549 cells. A549 cells were infected for 8, 16, 24, or 48 h with RSV mKate2-BirA*-Flag or RSV NS1-BirA*-Flag at an MOI of 1, 0.5, 0.1, or 0.01, respectively. At all time points, MED25 could be coimmunoprecipitated by NS1-BirA*-Flag (Fig. 5G). These results highlight that MED25 specifically interacts with NS1 during an RSV infection. The apparent upregulation of MED25 by RSV infection, as observed at 16 and 24 h postinfection (hpi) with RSV NS1-BirA*-Flag or RSV mKate2-BirA*-Flag (Fig. 5G, right panel), was not observed in repeat experiments.

### Replication of RSV B1 is significantly enhanced in MED25 knockout A549 cells.

**FIG 6:**
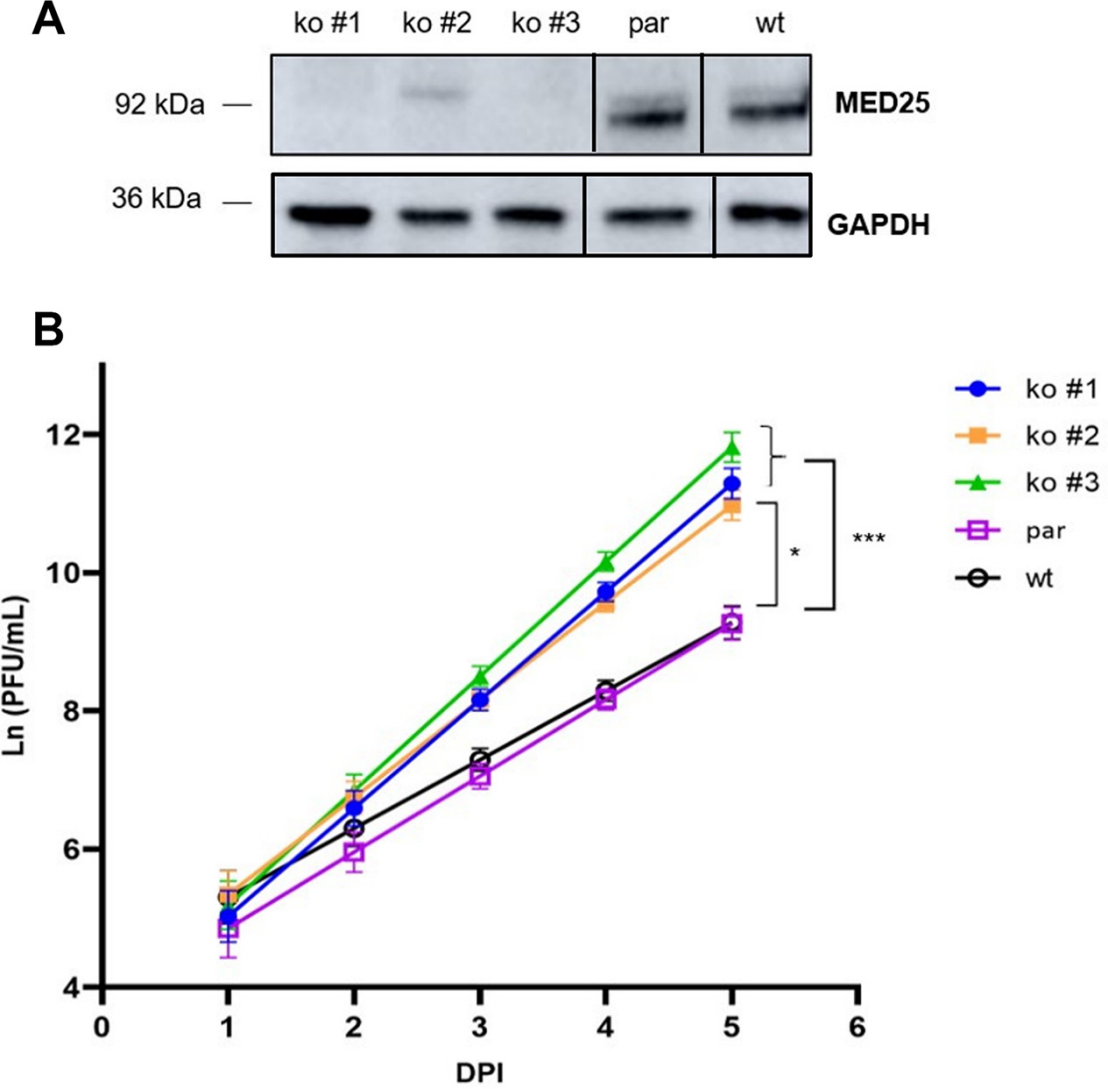
Replication of RSV B1 is significantly enhanced in MED25 knockout A549 cells. (A) The lack of expression of MED25 protein in knockout cells was analyzed by Western blotting using an anti-MED25 antibody. GAPDH staining was used as loading control. (B) MED25 knockout (ko #1, ko #2, and ko #3), parental (par), and wild-type (wt) A549 cells were infected with RSV B1 (MOI, 0.005). For 5 days postinfection, supernatant was collected daily for virus titration in wild-type A549 cells by using plaque assays. PFU/mL data from two independent experiments, each performed in triplicate, were analyzed using a generalized linear mixed model (see “Statistical analysis of replication kinetics”). The scale of the *y* axis is based upon a natural logarithm. ***, *P* < 0.005; *, *P* < 0.05.

To examine the role of MED25 during an RSV infection, we generated three MED25 knockout A549 cell clones (ko #1, ko #2 and ko #3) using CRISPR/Cas9 endonuclease-mediated genome editing. A549 cells were the cell line of choice, because these cells are IFN competent and permissive for RSV (17, 51, 52). As a control, we transfected A549 cells with the parental Cas9 expression vector without genomic RNA (gRNA), which are further referred to as parental A549 cells. In ko #1 and ko #3, MED25 expression was undetectable, while in ko #2, residual expression of a possible MED25 isoform was detectable (Fig. 6A). The 5 different A549 cell lines (ko #1 to ko #3, parental, and wild type) were infected with laboratory strain RSV B1 at an MOI of 0.005 (based on Vero cell titers), and supernatant was collected daily until 5 days postinfection for virus titration in wild-type A549 cells by plaque assay. In all three MED25 knockout A549 cell clones, a significantly increased viral titer was observed compared to that of the parental and wild-type A549 cells, suggesting that MED25 exerts an antiviral effect during an RSV infection (Fig. 6B and Fig. S6A). Similarly, the laboratory strain RSV A2 replicated to higher viral titers in the MED25 knockout A549 cell clones than in the parental and wild-type A549 cells, although the difference was less pronounced than with RSV B1 (Fig. S5 and Fig. S6B).

## DISCUSSION

Since viruses have a limited coding capacity, they must rely on the host cellular machinery to complete their infection cycle. Besides, viruses need to evade the host defense system to successfully complete their replication. A better understanding of the RSV-host protein interactions might reveal cellular networks that are hijacked by this virus. The identification of host factors required for RSV replication is important in the development of potential host-targeting antivirals. To obtain a comprehensive overview of the cellular interacting partners of RSV NS1, we performed three complementary protein-protein interaction screens, i.e., BioID, MAPPIT, and KISS. The BioID screen was performed with a recombinant mKate2 reporter RSV in which BirA* was fused in frame with NS1 in the viral genome. This allowed us to investigate candidate NS1-interacting proteins during an RSV infection in a relevant cell line (A549 cells). We first showed that the rescued RSV NS1-BirA*-Flag virus and the RSV mKate2-BirA*-Flag control virus can biotinylate proteins in the proximity of their corresponding BirA* fusion protein and are therefore convenient for use in a BioID screen. This screen revealed the presence of several subunits of the Mediator complex in the proximity of NS1, i.e., MED1, MED15, MED25, and MED27. These findings are in line with three reported proteomics studies that made use of overexpressed tagged NS1 that also identified several Mediator subunits as interactors of NS1, including MED25 (22, 23, 50). Mediator is a large complex that is involved in the transduction of signals between transcription factors and the RNA polymerase II machinery (53–55). As such, the Mediator complex regulates transcription of specific genes, including antiviral genes. MED23, for example, induces IFN-λ through direct interaction with the IRF7 transcription factor (56). Through this mechanism, MED23 can counteract the replication of herpes simplex virus I.

To catalogue the interactome of NS1 comprehensively, we performed two additional screens using the MAPPIT and KISS technology. MED25, a subunit of the Mediator complex, was identified in all three performed screens as a candidate NS1-interacting protein. Using coimmunoprecipitation in HEK293T cells, we could further confirm the interaction between NS1 and MED25. More specifically, the ACID domain of MED25 is targeted by NS1. Interestingly, after infection of A549 cells with RSV NS1-BirA*-Flag, we could coimmunoprecipitate endogenous MED25 with NS1-BirA*. Overall, we not only confirmed the interaction between NS1 and MED25 upon overexpression of NS1 alone but also identified this interaction during an RSV infection. This is particularly of interest since NS2, another IFN-antagonizing protein expressed during an RSV infection, interacts with NS1 and is thought to target NS1 to the mitochondria (13, 19). Our data confirm that NS1 interacts with MED25 during an RSV infection and that a fraction of NS1 localizes to the nucleus.

We were thus able to confirm the interaction between NS1 and a subunit of the Mediator complex in various ways. Mediator subunits are recruited by transcription factors to promoters of their target genes. Subsequently, the assembly of the RNA polymerase II preinitiation complex can occur on these gene promoters (57). As such, the NS1-MED25 interaction suggests a potential role of NS1 in transcription regulation.

Recently, Pei et al. reported that RSV NS1 colocalizes with Mediator and binds to regulatory elements near differentially expressed genes upon RSV infection, including the IFIT2 viral immune response gene, to inhibit their transcription (22). Moreover, these authors showed that the C-terminal α3 helix of NS1 plays a crucial role in the modulation of the host gene transcription. Here, we demonstrate that the α3 helix of NS1 is required for the interaction with MED25 (Fig. 5F). In MED25 knockout A549 cells, an RSV B1 strain replicated significantly faster than it did in control A549 cells. Enhanced replication of the RSV A2 strain that we tested was also observed, although in this case, the difference with MED25-expressing control cells did not reach statistical significance. The amino acid sequence of NS1 is well conserved (87%) between the RSV B1 and RSV A2 strains (see Fig. S7 in the supplemental material). The amino acid sequence of NS1 of RSV A2-line19F is identical to that of RSV A2. There are 5 amino acid residue differences (at positions 121, 129, 131, 135, and 139) between the NS1 α3 helix of RSV A2 and RSV B1 (Fig. S7). Possibly, these differences in NS1 allow RSV B1 to interfere more potently with MED25 function.

In our screens, we also identified IFIT2 as a candidate interaction partner of NS1, suggesting that NS1 affects IFIT2 at two levels: by suppressing its transcription and, potentially, by directly interacting with IFIT2, thereby hindering the antiviral effect of IFIT2. A recent study demonstrated that RSV is restricted in an IFIT2-dependent manner, as silencing or overexpression of IFIT2 in HEp2 cells resulted in an increased or reduced replication rate, respectively, of RSV (58).

The interaction of viral proteins with the host protein MED25 has been reported earlier for herpesviruses: herpes simplex virus I VP16 targets MED25 to regulate transcription of its viral genes (45–47, 59). The transactivation domain of IE62 from varicella-zoster virus interacts with MED25, resulting in increased transactivation activity of IE62 (60). It has also been suggested that Lana-1 of Kaposi’s sarcoma-associated herpesvirus (KSHV) contributes to KSHV-induced transcriptional reprogramming by acting as an adaptor between MED25 and specific transcription factors (61). The interaction between these viral proteins and MED25 is suggested to favor viral replication through activation of transcription of the viral genes. In contrast, using MED25 knockout A549 cells, we showed that MED25 potentially possesses an antiviral effect (Fig. 6B). This is in line with the study of Pei et al., who demonstrated that RSV NS1 interacts with Mediator and gene regulatory elements to modulate host gene transcription as a way to counteract host antiviral mechanisms (22).

We also identified proteins that were in common in at least two of the three performed protein-protein mapping screens, including many proteins with an E3 ubiquitin ligase activity. It has been reported that NS1 interacts with elongin C and cullin-2, allowing NS1 to act as a scaffold on which an E3 ligase complex can be composed (12). In addition, Goswami et al. proposed the existence of an NS degradasome (NSD) complex, in which NS1 and NS2 work cooperatively to degrade multiple key IFN signaling molecules (RIG-I, IRF3, IRF7, STAT2, etc.) (13). It is hypothesized that the NSD complex also comprises several host factors, but the exact composition still needs to be determined. Therefore, it is tempting to speculate that some of the ubiquitin ligase-associated proteins that we have identified as NS1-interacting proteins are associated with the NSD, providing a manner for NS1 to enhance polyubiquitination of signaling proteins of the innate immune system.

In summary, we demonstrate an interaction between NS1 and the Mediator subunit MED25 for the first time during an RSV infection. We also provide evidence that in MED25 knockout A549 cells, the replication of at least some RSV strains is enhanced, suggesting a potential antiviral role of MED25 during RSV infection. Mediator consists of a complex of transcriptional coactivators, suggesting that NS1 targets MED25 to disturb host transcription and to reshape the host immune response to infection.

## MATERIALS AND METHODS

### Cell culture.

HEK293 T cells (a gift from M. Hall, University of Birmingham, Birmingham, UK), Vero cells (ATCC, CCL-81), HEp-2 cells (ATCC, CCL-23), HeLa cells (ATCC, CCL-2), and A549 cells (ATCC, CCL-185) were grown in Dulbecco’s modified Eagle’s medium (DMEM) supplemented with 10% heat-inactivated fetal calf serum (FCS), 2 mM l-glutamine, and 1 mM sodium pyruvate at 37°C in the presence of 5% carbon dioxide. BSR-T7/5 cells (a gift from Peter Delputte, University of Antwerp, Antwerp, Belgium) were cultured in the same medium as mentioned above but supplemented with 1 mg/mL G418 to maintain the T7 polymerase transgene.

### Recovery of recombinant RSV NS1- BirA*-Flag and RSV mKate2-BirA*-Flag from cDNA.

To generate wild-type recombinant RSV and BirA (the R118G mutant is designated BirA* [25])-Flag-expressing RSVs, we used BAC pSynkRSV-line19F (obtained from the NIH Biodefense and Emerging Infections Research Resources Repository, NIAID, NIH; BEI Resources product NR-36460), which contains the RSV cDNA from strain RSV A2-line19F (31). To generate BirA*-Flag-containing RSVs, we modified the BAC by Red/ET recombination (62), which allowed us to fuse the BirA*-Flag sequence C-terminally in-frame with the NS1 open reading frame (NS1-BirA*-Flag BAC). For this, we transformed the pSynkRSV-line19F BAC into E. coli DH10B bacteria together with the recombination plasmid pSC101-BAD-γβαA-tet (a gift from A. Francis Stewart, TU Dresden). After activation of the recombination plasmid by 10% l-arabinose, the recombination fragment was introduced and positive clones were selected by kanamycin selection. The recombination fragment consisted of the BirA*-Flag sequence, followed by a floxed kan/neo resistance cassette (obtained from GenScript) and was flanked by a 50-bp sequence homologous to the BAC. It was generated by amplifying the BirA*-Flag-Lox-kan/neo-LoxP sequence by PCR (*Pfu* DNA polymerase from Promega) using primers containing 50-bp homology to the BAC up- and downstream of the insertion site. The selectable floxed kan/neo cassette was subsequently removed by transforming the modified BAC into SW106 bacteria expressing Cre recombinase upon induction with 10% l-arabinose.

For the generation of the control RSV cDNA with BirA*-Flag fused C-terminally to mKate2 (mKate2-BirA*-Flag BAC), two long BAC-derived fragments were generated by PCR using KAPA HiFi HotStart ReadyMix (Roche), followed by an assembly with the BirA* insert using the NEBuilder hi-fi DNA assembly master mix (New England Biolabs; E2621S). Recombinant RSV expressing NS1-BirA*-Flag or mKate2-BirA*-Flag was obtained by transfection of the corresponding engineered RSV cDNA into BSR-T7/5 cells as described previously (30).

### Viruses.

The laboratory strains RSV B1 (ATCC VR1580) and RSV-A2 (ATCC VR1540) were propagated on HEp-2 cells and quantified on Vero cells by plaque assay using goat anti-RSV serum to stain plaques (AB1128; Chemicon International). The generated RSV NS1-BirA*-Flag and RSV mKate2-BirA*-Flag were propagated on HEp-2 cells, and their infectivity was quantified on A549 cells by plaque assay.

### Sequencing of the recombinant RSVs.

Viral stocks of RSV mKate2, RSV NS1-BirA*-Flag, and RSV mKate2-BirA*-Flag were subjected to metagenomics sequencing using Oxford Nanopore Technologies (ONT) long-read sequencing at PathoSense BV (63). The obtained sequence reads were used to assemble the viral genomes using canu (v.2.2) (64), minimap2 (v.2.24) (65), and medaka (v.1.6.0; ONT) software packages. The *de novo*-assembled full-genome sequences were identical to the corresponding BAC sequences that were used to rescue the viruses, except for the following mutations: a silent mutation in the M2-2 protein (corresponding to amino acid position 45) and one mutation in the noncoding region (nucleotide position 5368) located between the SH and G genes for RSV mKate2; a silent mutation in the M2-2 protein (corresponding to amino acid position 45), a nonsynonymous mutation in the F protein (L142V), and one mutation in the noncoding region (nucleotide position 6506) located between the SH and G genes for RSV NS1-BirA*-Flag; and two nonsynonymous mutations in the F protein (V144A and A177V), one mutation in the noncoding region (nucleotide position 6421) between the SH and G genes, and one mutation in the noncoding region (nucleotide position 5052) between the P and M genes for RSV mKate2-BirA*-Flag.

### Immunofluorescence microscopy.

A549 cells were seeded in six-well plates. The next day, cells were infected with RSV mKate2, RSV mKate2-BirA*-Flag, or RSV NS1-BirA*-Flag at different MOIs (0.1, 1, and 2.5) or were mock infected for 4 h at 37°C. After 4 h, virus inoculum-containing medium was replaced with DMEM supplemented with 2% fetal bovine serum (FBS) and 50 μM biotin. At 12, 20, or 28 hpi, the cells were fixed with 2% paraformaldehyde and stained with streptavidin-AF488 and Hoechst stain. Immunofluorescent pictures were taken with an Olympus CKX53 microscope combined with the ToupView software.

### Confocal microscopy of RSV mKate2-BirA* and RSV NS1-BirA*.

A549 cells were seeded on μ-slide 8-well coverslips (Ibidi) and infected with RSV mKate2-BirA*-Flag or RSV NS1-BirA*-Flag at an MOI of 1 or were mock infected for 4 h at 37°C. After 4 h, the virus inoculum-containing medium was replaced with DMEM supplemented with 2% FBS and 50 μM biotin for an additional 20 h. The cell medium was subsequently removed, and the cells were fixed with 4% paraformaldehyde and permeabilized with 0.2% Triton X-100. The cells were blocked and stained with streptavidin-AF488. Nuclei were stained with 4′,6-diamidino-2-phenylindole (DAPI). Images were acquired using a Zeiss LSM 880 Fast Airyscan.

### BioID proxeome screen.

Five million A549 cells were seeded in 14-cm petri dishes. The next day, eight petri dishes were infected with RSV NS1-BirA*-Flag, eight petri dishes were infected with RSV mKate2-BirA*-Flag, four petri dishes were mock infected, and four petri dishes were infected with wild-type mKate2 expressing RSV at an MOI of 1 for 4 h at 37°C. After 4 h, virus-containing medium was replaced with DMEM supplemented with 2% FBS and 50 μM biotin. After 16 h (20 h after infection), the biotin-containing medium was removed and the cells were gently washed with ice-cold phosphate-buffered saline (PBS) and collected in PBS by scraping the cells from the petri dish. Cells from two petri dishes were pooled to form one biological sample. The cells were pelleted by centrifugation at 500 × *g* for 5 min and washed with PBS. After another centrifugation at 500 × *g* for 5 min, the supernatant was removed and cell pellets were stored at −20°C. Cell pellets were thawed on ice and resuspended in 1 mL lysis buffer (50 mM Tris-HCl [pH 7.5], 150 mM NaCl, 1% NP-40, 1 mM EDTA, 1 mM EGTA, 0,5% sodium deoxycholate, 0.1% SDS, and complete protease inhibitor cocktail [04693132001; Sigma-Aldrich]). A total of 210 U benzonase nuclease (E1014; Sigma-Aldrich) was added to the lysates, which were then incubated at 4°C for 1 h with agitation. Cell lysates were subsequently sonicated at 30% amplitude (3 intervals of 9 s of sonication/5 s of rest) and centrifuged at 4,332 × *g* for 15 min at 4°C to remove the insoluble fraction. Meanwhile, streptavidin-Sepharose high-performance beads (17-5113-01; GE Healthcare Life Sciences) were washed three times with 20 bed volumes of lysis buffer without sodium deoxycholate and protease inhibitors. Washed beads were added to each sample and incubated for 3 h at 4°C under constant rotation. After 3 h, the beads were pelleted by centrifugation at 400 × *g* for 1 min. The supernatant was removed, and the beads were washed three times in 1 mL lysis buffer without sodium deoxycholate and protease inhibitors. The beads were subsequently washed three times with trypsin digestion buffer (20 mM Tris-HCl [pH 8.0] and 2 mM CaCl_2_) and finally resuspended in 150 μL trypsin digestion buffer.

### Sample preparation and LC-MS/MS.

Washed beads were incubated for 4 h with 1 μg of trypsin (Promega) at 37°C. After removal of the beads, another 1 μg of trypsin was added and proteins were further digested overnight at 37°C. Peptides were acidified with trifluoroacetic acid (TFA) to lower the pH below 3 and desalted on reversed-phase C_18_ OMIX tips (Agilent). The tips were first washed three times with 100 μL prewash buffer (0.1% TFA in water-acetonitrile [ACN; 20:80, vol/vol]) and preequilibrated five times with 100 μL wash buffer (0.1% TFA in water) before the sample was loaded on the tip. After peptide binding, the tip was washed three times with 100 μL of wash buffer and peptides were eluted twice with 100 μL elution buffer (0.1% TFA in water/ACN [40:60, vol/vol]). The combined elution mixtures were dried in a vacuum concentrator and redissolved in 20 μL loading solvent A (0.1% TFA in water-ACN [98:2, vol/vol]) of which 0.5 μL was injected for LC-MS/MS analysis on an Ultimate 3000 RSLCnano system (Thermo Fisher Scientific) in-line connected to a Q Exactive mass spectrometer (Thermo Fisher Scientific) equipped with a Pneu Nimbus dual ion source (Phoenix S&T). Trapping was performed at 10 μL/min for 4 min in loading solvent A on a 20-mm trapping column (made in-house, 100 μm internal diameter [i.d.], 5-μm beads; C_18_ ReproSil-HD; Dr. Maisch) and the sample was loaded on a 50-cm μPAC column with C_18_-end-capped functionality (PharmaFluidics) mounted in the Ultimate 3000’s column oven and kept at a constant temperature of 50°C. For proper ionization, a fused silica PicoTip emitter (10 μm i.d.; New Objective) was connected to the μPAC outlet union and a grounded connection was provided to this union. Peptides were eluted by a nonlinear increase from 1% to 50% MS solvent B (0.1% formic acid (FA) in water-ACN [2:8, vol/vol]) over 91 min, first at a flow rate of 750 nl/min and then at 300 nl/min, followed by a 9-min wash reaching 95% MS solvent B and reequilibration with MS solvent A (0.1% FA in water).

The MS was operated in a data-dependent, positive ionization mode, with automatic switching between MS and MS/MS acquisition for the five most abundant peaks in a given MS spectrum. One MS1 scan (*m/z* 400 to 2,000, automatic gain control [AGC] target of 3E6 ions, maximum ion injection time of 80 ms), acquired at a resolution of 70,000 (at 200 *m/z*), was followed by up to five MS/MS scans (resolution 17,500 at 200 *m/z*) of the most intense ions fulfilling predefined selection criteria (AGC target of 5E4 ions, maximum ion injection time of 80 ms, isolation window of 2 Da, fixed first mass of 140 *m/z*) (spectrum data type: centroid, intensity threshold of 1.3E4, exclusion of unassigned, 1, 5 to 8, and >8 positively charged precursors, peptide match preferred, exclude isotopes on, dynamic exclusion time of 12 s). The higher energy collision dissociation (HCD) collision energy was set to 25% normalized collision energy, and the polydimethylcyclosiloxane background ion at 445.12002 Da was used for internal calibration (lock mass). QCloud was used to control instrument longitudinal performance during the project (66).

### Data analysis of BioID proteomics.

Data analysis was performed with MaxQuant (version 2.0.1.0) using the Andromeda search engine with default search settings, including a false discovery rate (FDR) set at 1% on peptide-spectrum matches (PSM) and protein level. The spectra of all 12 LC-MS/MS runs were interrogated against the human proteins in the Swiss-Prot Reference Proteome database (database release version of January 2021 containing 20,621 human protein sequences [https://www.uniprot.org]), the HRSV-A proteins in the UniProt database (release version of July 2021 containing 7,972 protein sequences [https://www.uniprot.org]), and the protein sequences of the NS1 and mKate constructs. The mass tolerances for precursor and fragment ions were set to 4.5 and 20 ppm, respectively, during the main search. Enzyme specificity was set as C-terminal to arginine and lysine, also allowing cleavage at proline bonds with a maximum of two missed cleavages. Variable modifications were set to oxidation of methionine residues and acetylation of protein N termini. Matching between runs was enabled with a matching time window of 0.7 min and an alignment time window of 20 min. Only proteins with at least one unique or razor peptide were retained, leading to the identification of 2,297 proteins. Proteins were quantified by the MaxLFQ algorithm integrated in the MaxQuant software. A minimum ratio count of two unique or razor peptides was required for quantification. Further data analysis was performed with the Perseus software (version 1.6.2.1) after the file of protein groups was loaded from MaxQuant. Reverse database hits were removed, label-free quantification (LFQ) intensities were log_2_ transformed, and replicate samples were grouped. Proteins with less than three valid values in at least one group were removed, and missing values were imputed from a normal distribution around the detection limit, leading to a list of 1,400 quantified proteins that were used for further data analysis. On these quantified proteins, a *t* test was performed for pairwise comparison of RSV NS1-BirA*-Flag-infected and RSV mKate2-BirA*-Flag-infected cells. The result of this *t* test is shown in the volcano plot in Fig. 2B. For each protein, the log_2_(NS1-BirA* RSV infected/mKate2-BirA* RSV infected) fold change value is indicated on the *x* axis, while the statistical significance (−log *P* value) is indicated on the *y* axis. Proteins outside the curved lines, set by an FDR value of 0.05 and an minimal fold change (So) value of 1 in the Perseus software, represent candidate NS1- or mKate2-interacting proteins. Three hundred forty-one proteins were significantly regulated and plotted in a heat map after nonsupervised hierarchical clustering. The mass spectrometry proteomics data have been deposited in the ProteomeXchange Consortium via the PRIDE partner repository with the data set identifier PXD034627.

### Bioinformatics analysis of BioID proteomics.

Qiagen IPA software (34) was used to analyze the cellular protein data set resulting from the BioID screen. The data set containing gene identifiers, corresponding *P* values, and differences (log_2_ fold change) was uploaded into the application. Each gene identifier was converted to its corresponding gene object in the Ingenuity Pathways Knowledge Base. The significantly enriched proteins were filtered in the Perseus software. The complete user data set was set as background, and an enrichment (core) analysis was performed by which molecular and cellular functions could be assigned to the proteins enriched in the RSV NS1-BirA*-Flag-infected cells. Additionally, a network analysis was performed in IPA in which all the genes of proteins were mapped against a global molecular network contained in the Ingenuity Pathways Knowledge Base, based on bundled information from literature and curated databases.

### MAPPIT screen.

For the construction of the MAPPIT bait vector, the coding sequence of RSV A2 NS1 was genetically fused in-frame to the C terminus of the EpoR–LR-F3 chimeric receptor in the pSEL + 2L vector, to generate pSEL + 2L − NS1. The construction of the preys, the primary MAPPIT screen, and the reporter reverse transfection mixture was performed as described previously (67, 68). MAPPIT retests were performed as described earlier (67, 69).

### KISS screen.

For the construction of the KISS bait vector, the coding sequence of RSV A2 NS1 was fused to the N terminus of a fragment of human TYK2 (amino acids 589 to 1187) and inserted into the pMet7 expression vector. The construction of the preys, the primary KISS screen, and retest screens was performed as described previously (27).

### Plasmid constructs.

By using Gateway cloning technology (Invitrogen), we generated an expression vector for MED25-Flag by transferring the MED25 cDNA sequence from the corresponding entry vector (pENTRY-MED25 vector, obtained from the human ORFeome library 8.1) into a destination vector for mammalian constitutive expression driven by the cytomegalovirus immediate early (CMV-IE) promoter and in-frame fusion with a C-terminal Flag tag.

The MED25-Flag expression vector was subsequently used to amplify the MED25-ACID domain (amino acids 394 to 543) by PCR, which was cloned into the same destination vector as described above, resulting in an expression vector for MED25 ACID-Flag. pcDNA5-Flag-MED25-VWA (expressing the MED25 VWA domain [amino acids 17 to 226]) was obtained from Addgene (plasmid no. 64772).

Codon-optimized NS1 (for mammalian expression), originating from RSV A2, was synthesized by Gen9 and cloned into the Gateway pDONR201 vector to generate an entry vector (pENTRY-NS1). The NS1 cDNA sequence from the corresponding entry vector was cloned using Gateway cloning into a destination vector for mammalian constitutive expression driven by the CMV-IE promoter and in-frame fusion with a C-terminal HA tag, resulting in an NS1-HA expression vector. This NS1-HA expression vector was used to generate the NS1 Y125A point mutant using a QuikChange site-directed mutagenesis kit (Stratagene), in accordance with the manufacturer’s instructions. The NS1 Δα3 mutant sequence (amino acids 1 to 118) was PCR amplified using the NS1-HA expression vector as a template. For the coimmunoprecipitation shown in Fig. 5F, the full-length and mutant NS1 sequences were cloned into the pcDNA 3.4 TOPO vector (A14697; Thermo Fisher Scientific) according to the manufacturer’s instructions.

The NS1-enhanced GFP (NS1-eGFP) expression plasmid was generated by introducing the RSV A2 NS1 coding DNA sequence (CDS) as an individual module part of a Golden Gate assembly cloning system available at the Eyckerman Lab (VIB-Center for Medical Biotechnology). This system is based on the constant enrichment of a library of different modules that contain different genetic elements (promoters, tags, linkers, coding sequences, etc.) and the assembly of those modules in destination vectors for mammalian expression in a single-step reaction based on BsaI digestion and ligation of complementary overhangs. RSV NS1 cDNA (from the pENTRY-NS1 plasmid) was amplified by PCR using primers with BsaI recognition sequence overhangs and was ligated into the destination vector with an EF1a promoter and C-terminally tagged with eGFP using a 3×G4S linker.

### Coimmunoprecipitation.

Three million HEK293T cells were seeded in 9-cm petri dishes. The following day, cells were transfected using polyethylenimine (PEI) (23966-1; Polysciences, Inc.) with 10 μg of the indicated constructs at a 1:5 ratio (5 μL of PEI [1 mg/mL] per μg of DNA). Briefly, DNA and PEI were diluted in Opti-MEM reduced serum medium and mixed for 10 min at room temperature (RT). DNA complexes were added dropwise to the HEK293T cells. Twenty-four hours after transfection, cells were lysed with lysis buffer (50 mM HEPES-KOH [pH 8.0], 100 mM KCl, 2 mM EDTA, 0.1% NP-40, 10% glycerol, 1 mM dithiothreitol [DTT], 0.5 mM phenylmethylsulfonyl fluoride [PMSF], 0.25 mM sodium orthovanadate, 50 mM glycerophosphate, 10 mM NaF, and protease inhibitor mix [Roche]). Lysates were incubated with mouse IgG1 anti-Flag-M2 antibody (F3165; Sigma-Aldrich) or with mouse anti-HA antibody (901533; BioLegend) overnight at 4°C under constant rotation. The next day, Flag or HA complexes were immunoprecipitated by the addition of Protein G Sepharose beads (17-0618-01; GE Healthcare) for 1 h at 4°C under constant rotation. Proteins that bound to the beads were detected by Western blot analysis.

### Western blot analysis.

Total cell lysates were boiled for 10 min at 99°C in 1× Laemmli buffer supplemented with 4.2% (vol/vol) β-mercaptoethanol. Proteins were separated by electrophoresis on precast 4 to 20% SDS-PAGE gels (Bio-Rad Laboratories) and semidry blotted on nitrocellulose membranes. Membranes were blocked with a 5% (wt/vol) lowfat milk solution in PBS. Proteins were detected with the following primary antibodies: rabbit anti-Flag (F7425; Sigma-Aldrich), rabbit anti-HA (ab9110; Abcam), and rabbit anti-MED25 (NBP2-55868; Novus Biologicals). As a control for equal loading, we used mouse anti-α-tubulin (T5168; Merck) or rabbit anti-glyceraldehyde-3-phosphate dehydrogenase (anti-GAPDH) (ab9485; Abcam). We used the following secondary antibodies: streptavidin-horseradish peroxidase (HRP) (RPN1231; Amersham Life Science), ECL donkey anti-rabbit IgG–HRP (NA934V, GE Healthcare), and ECL sheep anti-mouse IgG–HRP (NA931V; GE Healthcare). Protein bands were visualized by using Pierce ECL Western blotting substrate (32106; Thermo Fisher Scientific) and a chemiluminescence imager (Amersham Imager 600; GE Healthcare).

### Generation of MED25 knockout A549 cells using CRISPR/Cas9.

The gRNA design tool Synthego was used to select two gRNAs targeting MED25: gRNA 1, TGTCCTCCCCTCCCAGTATG, and gRNA 2, GTACTGGGTCCCCCCATACT. These gRNAs were cloned in the pSpCas9 (BB)-2A-GFP vector (Addgene plasmid 48138) according to the standard assembly protocol (70). A549 cells were transfected with pSpCas9(BB)-2A-eGFP plasmids containing the MED25 gRNA duplex using Lipofectamine LTX with PLUS reagent (Invitrogen/Life Technologies). To generate a control A549 cell line (further referred to as parental A549 cell line), we transfected A549 cells with the parental Cas9 vector without gRNA. Forty-eight hours posttransfection, GFP-positive cells were sorted as single cells in a 96-well plate by fluorescence-activated cell sorting (FACS). Single-cell clones were further verified for MED25 knockout by genomic DNA sequencing and immunoblotting (Fig. 6A).

### Replication kinetics of RSV in MED25 knockout A549 cells.

Confluent monolayers of MED25 knockout A549 cells in flat-bottom 96-well plates were infected in triplicate at an MOI of 0.005 with RSV B1, RSV A2, RSV MAD/GM2_2/12, or RSV B49 in Opti-MEM reduced serum medium for 4 h at 37°C. After 4 h, the RSV inoculum was replaced by DMEM supplemented with 2% FCS and 2 mM l-glutamine. Until 5 days postinfection, 20 μL of supernatant was harvested daily and mixed with 20 μL of Hanks’ balanced salt solution supplemented with 40% sucrose, snap-frozen in liquid nitrogen, and stored at −80°C. For each replicate, the titer was further determined in duplicate in a plaque assay. For the plaque assay, wild-type A549 cells were seeded in 96-well plates and incubated with serial dilutions of virus samples for 4 h at 37°C. After 4 h, virus inoculum was replaced with DMEM supplemented with 2% FCS, 2 mM l-glutamine, and 0.6% (wt/vol) Avicel RC-851 (FMC Biopolymers) and incubated for 3 days at 37°C. The culture medium was removed, and cells were fixed with 4% paraformaldehyde in PBS for 20 min at RT. Cells were then washed two times with PBS and permeabilized with 0.2% Triton X-100 for 10 min at RT. Cells were blocked with 1% bovine serum albumin (BSA; A4503, Sigma-Aldrich) in PBS for at least 1 h. RSV plaques were stained with a polyclonal goat anti-RSV serum (AB1128; Chemicon International) diluted 1:1,000 in 0.5% BSA in PBS for 2 h at RT and a horseradish peroxidase-conjugated anti-goat IgG (Sc-2020; Santa Cruz Biotechnology, Inc.) diluted 1:3,000 in 0.5% BSA in PBS for 1 h at RT. After each antibody inoculation, cells were washed four times with PBS. Plaques were visualized by use of TrueBlue peroxidase substrate (5510; Sera Care) and washed with water when the plaque intensity was satisfactory. The plaques were counted, and the number of PFU/mL was calculated.

### Statistical analysis of replication kinetics.

The experiment was laid out in three complete blocks with two replicates. Measurements were taken daily for 5 days. A generalized linear mixed model (GLMM), as implemented in Genstat v21 (71), was fitted to the count data, with a log link function *g*(μ) and assuming a gamma distribution for random effects. The fixed model can be written as replicate + replicate.block + genotype + time + genotype.time, with time as a variable. Two random terms accommodate for well-to-well variation over time and pseudoreplication (two observations from the same well), with time as a factor. The dispersion parameter for the variance of the response was estimated from the residual mean square of the fitted model. *t* statistics were used to assess the significance of genotype and genotype by time effects (on the log-transformed scale) by pairwise comparisons to the wild type and of wild type by time reference levels.

### Colocalization analysis by confocal microscopy.

HeLa cells were seeded on μ-slide 8-well coverslips (Ibidi) and transfected with NS1-GFP (100 ng per well) using FuGENE in accordance with the manufacturer’s instructions. The next day, the cell medium was removed and the cells were fixed with 4% paraformaldehyde and permeabilized with 0.2% Triton X-100. The cells were blocked and immunostained with an antibody specific for MED25 (Atlas Antibodies; HPA068802) and Alexa Fluor-568 conjugated secondary antibody. Nuclei staining was performed using DAPI. Images were acquired using a Zeiss LSM 880 Fast Airyscan.

### Data availability.

The mass spectrometry proteomics data have been deposited in the ProteomeXchange Consortium via the PRIDE partner repository under the data set identifier PXD034627. The output sequencing reads of the recombinant viruses have been submitted to NCBI GenBank and are available under accession numbers OP242455 (RSV mKate2), OP242456 (RSV mKate2-BirA*-Flag), and OP242457 (RSV NS1-BirA*-Flag).
