# Supplementary material for: An Unexpected Encounter: Respiratory Syncytial Virus Nonstructural Protein 1 Interacts with Mediator Subunit MED25
Source: J Virol. 2022 Sep 14;96(19):e01297-22. doi: 10.1128/jvi.01297-22 (PMC9555202; doi:10.1128/jvi.01297-22)
Supplement: Supplemental file 1 — Fig. S1 to S7, Tables S1, S4, and S5, and description of Tables S2 and S3. Download jvi.01297-22-s0003.pdf, PDF file, 0.8 MB [file jvi.01297-22-s0003.pdf]

Supplementary materials for

An Unexpected Encounter: Respiratory Syncytial Virus Non-Structural Protein Interacts With Mediator Subunit MED25

Van Royen *et al*.

This pdf file includes:

Supplementary figures 1-7

Supplementary tables S1, S4, and S5

We note that supplementary tables S2 and S3 are available as separate excel files

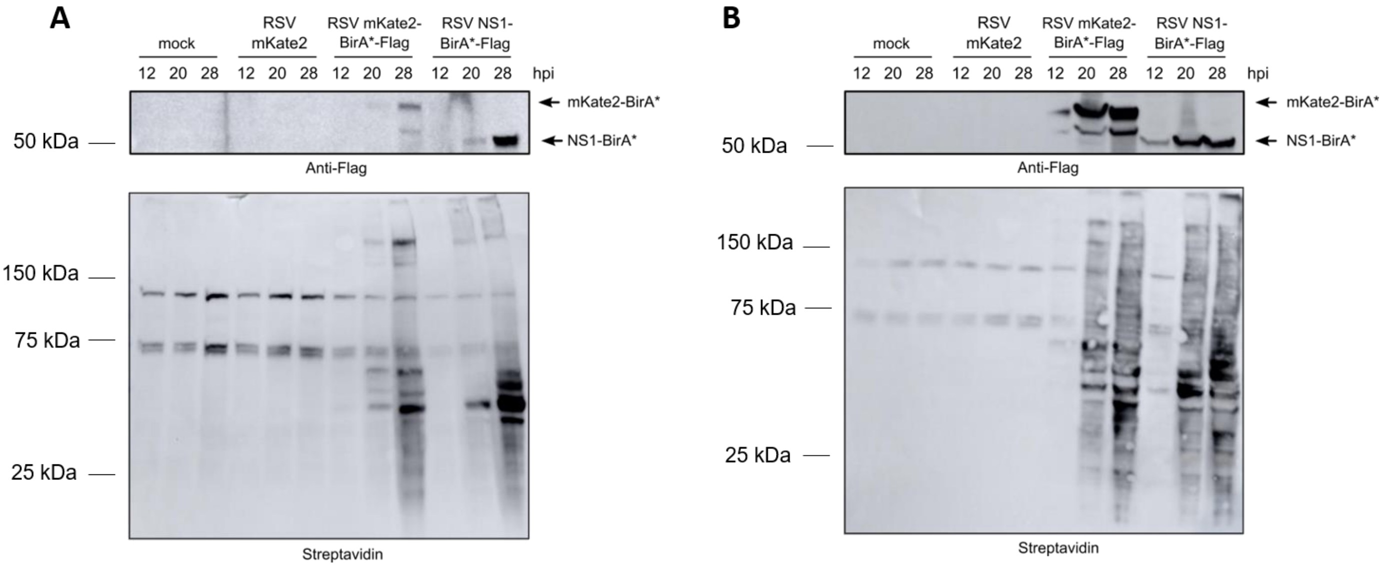

Supplementary Figure 1. Characterization of RSV NS1-BirA*-Flag and RSV mKate2-BirA*-Flag viruses after infection of A549 cells with a MOI of 0.1 (A) or 2.5 (B). Immunoblots show expression of NS1-BirA*- and mKate2-BirA*-Flag fusion proteins (upper panel) and biotinylation capacity upon expression of the fusion proteins (lower panel). A549 cells were infected with the recombinant BirA* expressing RSVs, the control RSV mKate2 (MOI = 0.1, results shown in (A) or MOI = 2.5, results shown in (B)), or mock-infected for 4h followed by incubation of the cells with 50 µM biotin for additional 8, 16 or 24h. Cell lysates were analyzed by western blotting with an anti-Flag antibody or streptavidin.

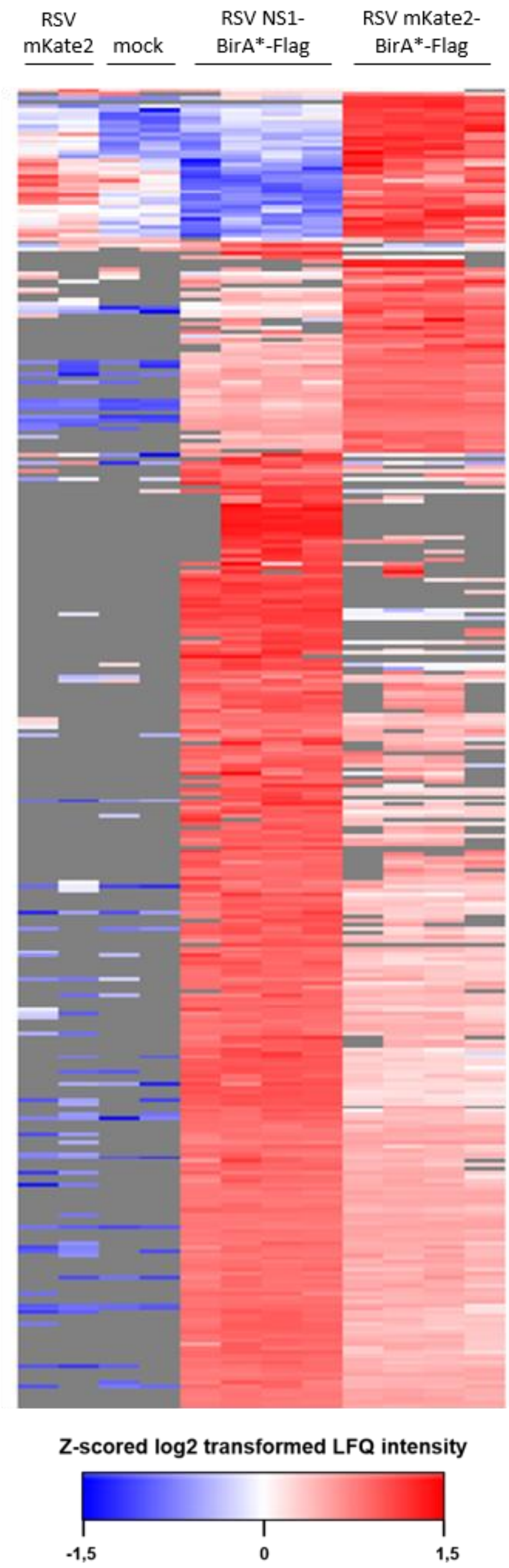

Supplementary Figure 2. A heatmap of the significant proteins as highlighted in Fig. 2B, where the imputed values are removed. Red indicates higher LFQ intensity, blue indicates lower LFQ intensity.

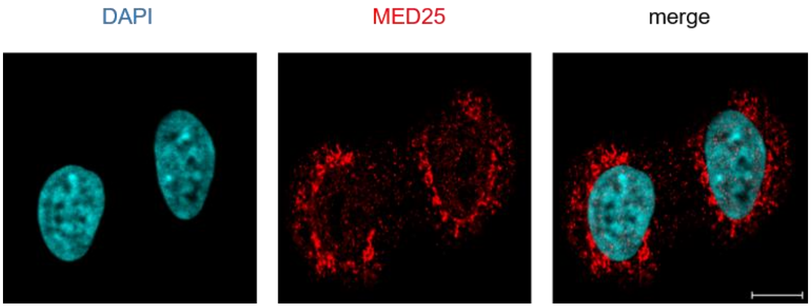

Supplementary Figure 3. Confocal micrographs of MED25 knockout A549 cells (ko #3) that were stained with anti-MED25 antibody (red) or DAPI (blue). Scale bar: 10 µm.

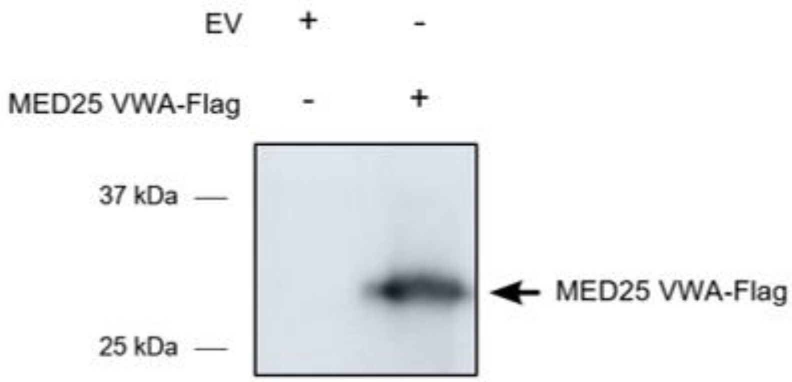

Supplementary Figure 4. HEK293T cells were transfected with a MED25 VWA-Flag expression vector or with an empty vector (EV). Blots were analyzed with an anti-Flag antibody.

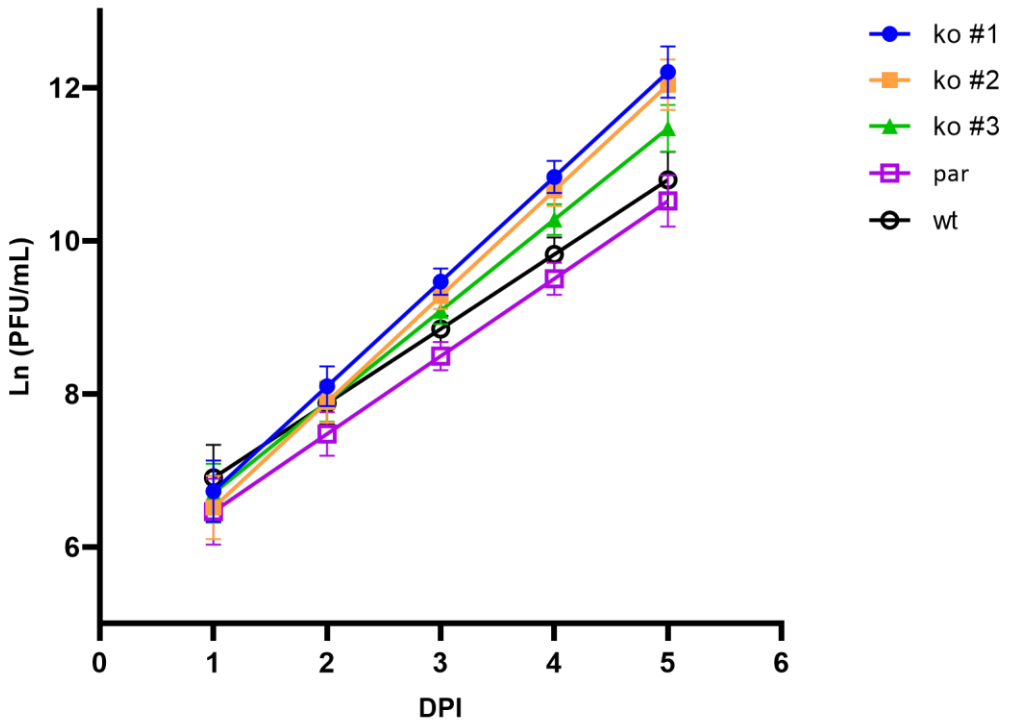

Supplementary Figure 5. **Replication of RSV A2 is enhanced in MED25 knockout A549 cells.** MED25 knockout (ko #1, ko #2 and ko #3), parental (par) and wild type (wt) A549 cells were infected with RSV A2 (MOI = 0.005). During 5 days post infection, supernatant was collected daily for virus titration in wild type A549 cells by plaque assay. PFU/ml data from two independent experiments, each performed in triplicate, were analyzed using a generalized linear mixed model (see Materials and Methods on statistics). The scale of the Y-axis is based on a natural logarithm.

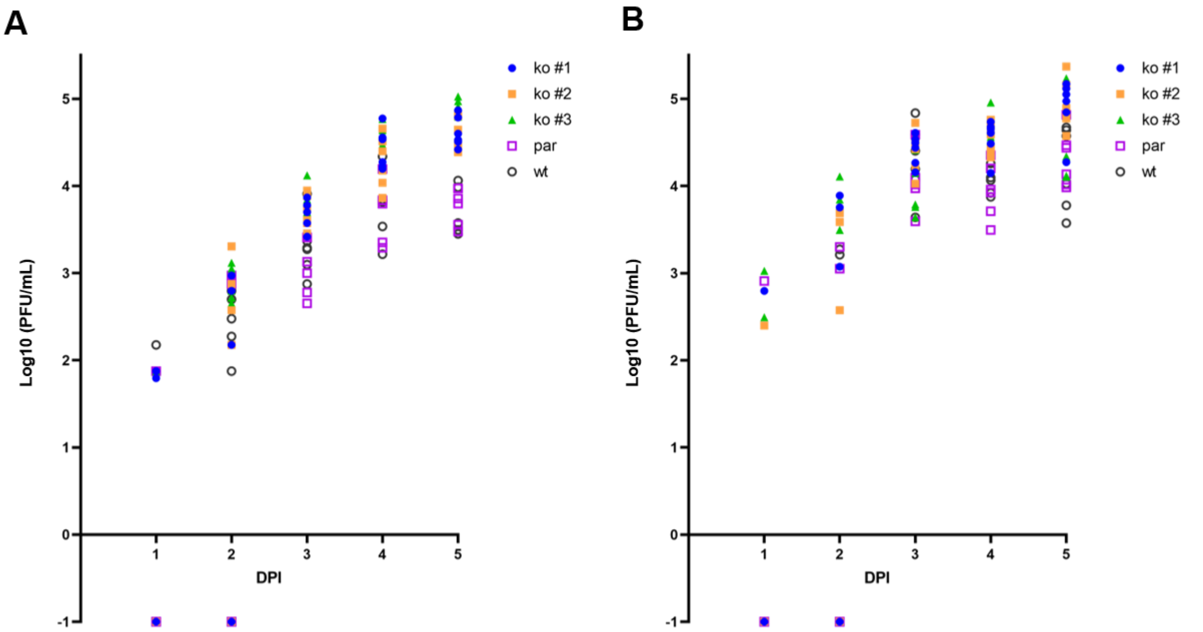

Supplementary Figure 6. Original data of the replication of RSV B1 (A) and RSV A2 (B) in MED25 knockout A549 cells. MED25 knockout (ko #1, ko #2 and ko #3), parental (par) and wild type (wt) A549 cells were infected with RSV B1 (A) or RSV A2 (B) (MOI = 0.005). During 5 days post infection, supernatant was collected daily for virus titration in wild type A549 cells by plaque assays. PFU/ml data from two independent experiments, each performed in triplicate are plotted.

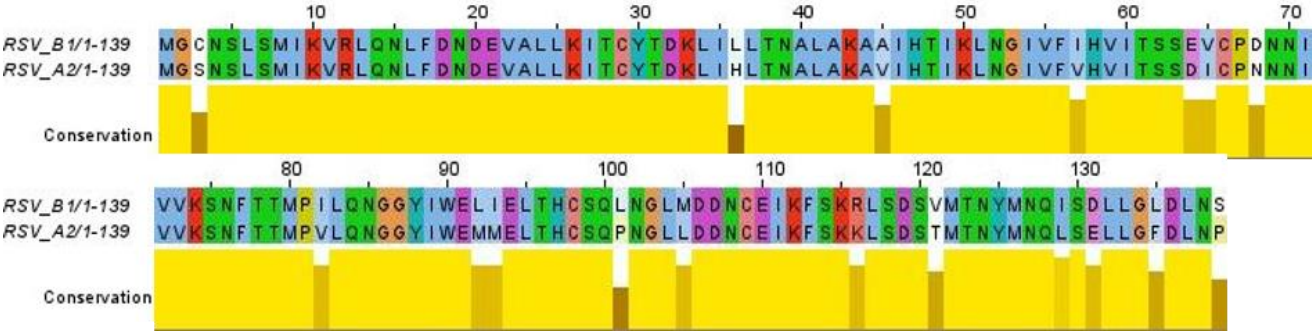

Supplementary Figure 7. Amino acid sequence alignment of NS1 of RSV B1 and RSV A2.

Supplementary Table S1. NS1 proxeome hits identified by BioID. Columns from left to right show the UniProt accession number, the gene name, the $\log_2$ (RSV NS1-BirA*-Flag RSV/ RSV mKate2-BirA*-Flag RSV) fold change value and -log p value as depicted in Fig. 2B and calculated as described under Materials and Methods.

Protein ID	Gene name	Log_2FC (RSV NS1-BirA*-Flag/ RSV mKate2-BirA*-Flag)	-log (P-value)
A0A060ACF2	RSV NS1	7.66	5.00
A0A088S9M1	RSV L	5.10	5.44
Q9BRX9	WDR83	3.99	4.67
Q9BW85	YJU2	3.27	4.80
P62316	SNRPD2	3.16	1.23
Q5VTR2	RNF20	3.11	4.76
Q9BTA9	WAC	3.01	3.92
P53680	AP2S1	2.94	3.24
O75150	RNF40	2.87	4.32
Q14004	CDK13	2.83	3.06
Q6P2C8	MED27	2.78	5.52
Q12873	CHD3	2.78	4.61
O00743	PPP6C	2.69	7.08
Q9NRL2	BAZ1A	2.65	1.80
O14879	IFIT3	2.60	4.98
Q96RN5	MED15	2.55	3.69
Q8N163	CCAR2	2.53	6.83
Q09161	NCBP1	2.51	2.78
P84090	ERH	2.47	1.32
Q9UPN7	PPP6R1	2.46	6.85
Q9Y261	FOXA2	2.44	1.45
P53999	SUB1	2.41	1.72
Q9P013	CWC15	2.37	2.27
Q00341	HDLBP	2.36	4.00
Q9UKZ1	CNOT11	2.34	4.10
O43237	DYNC1LI2	2.33	2.24
Q8NB46	ANKRD52	2.32	7.07
Q5H9R7	PPP6R3	2.22	7.83
Q9Y5K5	UCHL5	2.22	2.51
P09913	IFIT2	2.20	3.30
Q5K651	SAMD9	2.19	2.72
Q9H0L4	CSTF2T	2.18	4.94
Q13618	CUL3	2.17	4.97
O43395	PRPF3	2.15	7.42
Q71SY5	MED25	2.12	1.41
P46013	MKI67	2.07	4.19

Q71DI3<br>Q16695<br>P84243<br>P68431<br>Q6NXT2	H3C15<br>H3-4<br>H3-3A<br>H3C4<br>H3-5	2.07	0.65
Q969G3	SMARCE1	2.06	5.73
O95777	LSM8	2.05	1.81
P33240	CSTF2	2.03	5.02
Q08379	GOLGA2	2.03	3.45
P17480	UBTF	2.01	2.62
P09936	UCHL1	1.99	3.22
Q9BVJ6	UTP14A	1.96	3.94
Q96ST3	SIN3A	1.92	4.47
Q12789	GTF3C1	1.92	3.43
Q9NVX0	HAUS2	1.91	2.12
O94913	PCF11	1.90	7.18
Q15427	SF3B4	1.90	5.24
Q13426	XRCC4	1.90	2.59
O15355	PPM1G	1.89	0.69
Q9NWX6	THG1L	1.89	5.42
Q9H0B6	KLC2	1.88	4.37
O95104	SCAF4	1.85	2.06
Q12800	TFCP2	1.84	2.37
Q8WUQ7	CACTIN	1.82	4.56
Q14839	CHD4	1.81	6.59
Q13123	IK	1.81	4.93
P62906	RPL10A	1.77	1.31
Q14254	FLOT2	1.77	1.23
Q5PRF9	SAMD4B	1.76	3.11
P51610	HCFC1	1.74	2.55
P78347	GTF2I	1.74	6.30
O43447	PPIH	1.73	2.18
Q9UK61	TASOR	1.73	3.58
Q15648	MED1	1.73	4.70
Q15907<br>P62491	RAB11B<br>RAB11A	1.72	2.17
Q9P1Z0	ZBTB4	1.71	1.32
Q92541	RTF1	1.70	1.52
P27540	ARNT	1.70	2.49
Q07666	KHDRBS1	1.70	2.02
Q8NFD5	ARID1B	1.69	9.93
Q9H307	PNN	1.68	3.68
Q8WXI9	GATAD2B	1.68	6.38
P17812	CTPS1	1.67	5.14
Q96I25	RBM17	1.66	4.91
Q6P2Q9	PRPF8	1.65	2.59
Q96NT0	CCDC115	1.65	1.68
P52739	ZNF131	1.63	2.65

P13984	GTF2F2	1.63	1.98
Q9BV36	MLPH	1.62	4.17
Q96K58	ZNF668	1.61	3.88
P33981	TTK	1.61	2.62
Q13330	MTA1	1.60	5.21
Q9BWJ5	SF3B5	1.60	1.30
Q9Y285	FARSA	1.60	2.74
O75717	WDHD1	1.59	5.62
P49189	ALDH9A1	1.58	2.72
O15294	OGT	1.58	5.41
O75376	NCOR1	1.57	2.17
P09914	IFIT1	1.56	5.00
Q9Y2H6	FNDC3A	1.56	4.31
Q13435	SF3B2	1.56	7.54
Q8NEN9	PDZD8	1.53	7.78
P49585	PCYT1A	1.53	1.74
Q96JH7	VCPIP1	1.53	2.79
Q8IXM2	BAP18	1.50	1.07
Q9UNH7	SNX6	1.49	1.13
P19532<br>P19484<br>O75030	TFE3<br>TFEB<br>MITF	1.49	4.00
Q8TDB6	DTX3L	1.47	8.33
Q13823	GNL2	1.47	4.24
Q15021	NCAPD2	1.47	1.53
Q99590	SCAF11	1.47	1.25
P18077	RPL35A	1.47	0.92
O94776	MTA2	1.46	3.71
P13861	PRKAR2A	1.46	1.21
Q9C0J8	WDR33	1.46	3.46
Q13185	CBX3	1.45	4.02
Q8IW35	CEP97	1.44	1.94
P49750	YLPM1	1.43	3.48
Q5JWF2	GNAS	1.43	4.01
Q1ED39	KNOP1	1.41	2.98
Q86UU0	BCL9L	1.41	5.13
Q8N511	TMEM199	1.41	1.06
Q96CS2	HAUS1	1.40	1.14
Q9H4A3	WNK1	1.40	1.12
Q14966	ZNF638	1.40	2.20
O95400	CD2BP2	1.40	1.16
Q9ULW0	TPX2	1.40	3.97
Q8IXH7	NELFCD	1.39	1.79
P42166	TMPO	1.39	4.42
P61011	SRP54	1.39	4.34
O95391	SLU7	1.37	1.51
Q9Y2H2	INPP5F	1.37	2.15
Q8IWI9	MGA	1.36	2.77

Q9NQB0<br>P36402<br>Q9UJU2	TCF7L2<br>TCF7<br>LEF1	1.36	1.22
Q9NSK0	KLC4	1.36	2.34
P17676	CEBPB	1.35	1.04
Q9Y5Q9	GTF3C3	1.35	1.12
O75153	CLUH	1.35	5.43
Q9NQG5	RPRD1B	1.35	5.04
Q96AG4	LRRC59	1.34	1.46
P33176	KIF5B	1.34	2.65
O15084	ANKRD28	1.33	7.49
Q9ULV3	CIZ1	1.32	5.05
Q9H9A5	CNOT10	1.32	5.26
P49915	GMPS	1.32	1.23
O15379	HDAC3	1.31	2.92
Q5VT52	RPRD2	1.30	5.62
O75909	CCNK	1.30	4.49
Q9H2M9	RAB3GAP2	1.28	1.50
Q13190	STX5	1.28	1.21
O75533	SF3B1	1.27	2.64
Q96HA1<br>A8CG34	POM121<br>POM121C	1.27	1.64
P86791<br>P86790	CCZ1<br>CCZ1B	1.26	3.65
Q9H501	ESF1	1.26	6.22
Q14980	NUMA1	1.26	2.30
Q02241	KIF23	1.25	5.13
P06748	NPM1	1.25	2.57
Q96EA4	SPDL1	1.24	7.28
Q12874	SF3A3	1.24	2.46
O43815	STRN	1.24	1.56
Q8N9Z2	CCDC71L	1.24	1.35
Q9Y2W1	THRAP3	1.24	3.67
P62136	PPP1CA	1.24	5.22
P35612	ADD2	1.24	1.45
Q9H2P0	ADNP	1.23	2.67
Q9UKL0<br>Q9P2K3	RCOR1<br>RCOR3	1.23	3.74
Q99459	CDC5L	1.23	3.13
O75182	SIN3B	1.23	1.91
Q9H869<br>Q3T8J9	YY1AP1<br>GON4L	1.21	2.51
O95239	KIF4A	1.20	3.82
Q9NX02	NLRP2	1.19	3.75
P13804	ETFA	1.19	1.15
Q8NI08	NCOA7	1.19	4.01
Q14686	NCOA6	1.19	4.53
P09661	SNRPA1	1.19	4.51

Q8IXQ6	PARP9	1.19	3.19
O43290	SART1	1.18	8.96
Q9Y3B4	SF3B6	1.18	4.02
Q8NHV4	NEDD1	1.17	2.67
O14776	TCERG1	1.16	3.36
Q86XA9	HEATR5A	1.16	4.50
Q9UMS4	PRPF19	1.15	5.33
O60264	SMARCA5	1.14	3.48
Q8IWZ8	SUGP1	1.14	5.16
Q8TAQ2	SMARCC2	1.13	1.46
Q15393	SF3B3	1.13	2.56
Q9Y618	NCOR2	1.12	5.02
P51991	HNRNPA3	1.12	1.53
O94830	DDHD2	1.12	1.96
O60341	KDM1A	1.11	3.72
Q8ND82	ZNF280C	1.11	1.85
P55265	ADAR	1.11	4.03
P35869	AHR	1.11	2.52
Q8TEQ6	GEMIN5	1.11	4.58
Q10570	CPSF1	1.10	2.83
Q96DI7	SNRNP40	1.10	2.10
Q2KHR3	QSER1	1.10	2.93
O00567	NOP56	1.09	3.15
Q9H3P7	ACBD3	1.08	3.89
Q6NZY4	ZCCHC8	1.08	4.10
O95163	ELP1	1.08	4.53
Q9BTC0	DIDO1	1.07	5.52
O43172	PRPF4	1.07	6.60
Q9NSI2	FAM207A	1.06	3.05
Q9NZM1	MYOF	1.06	2.42
P53621	COPA	1.05	4.80
Q13547	HDAC1	1.05	4.03
Q9P2N5	RBM27	1.04	3.84
P00338	LDHA	1.04	4.14
Q9UKX7	NUP50	1.03	1.79
O94842	TOX4	1.03	7.84
P50750	CDK9	1.03	4.00
Q13573	SNW1	1.02	4.09
P50613	CDK7	1.02	3.61
O75400	PRPF40A	1.02	3.48
Q8IX01	SUGP2	1.01	4.14
Q86VM9	ZC3H18	1.00	1.97
Q9UPT8	ZC3H4	1.00	1.73
Q9UIG0	BAZ1B	0.99	5.74
Q9UFC0	LRWD1	0.99	5.67
O15042	U2SURP	0.99	5.71
Q9UJV9	DDX41	0.98	3.71
Q13263	TRIM28	0.98	3.78

Q96GX5	MASTL	0.98	2.60
Q92989	CLP1	0.97	2.14
A0A088STS5	RSV M2-1	0.97	4.23
Q5VZ89	DENND4C	0.96	6.25
Q7L014	DDX46	0.95	5.21
Q9BZK7	TBL1XR1	0.95	4.43
O60885	BRD4	0.94	4.35
Q15029	EFTUD2	0.94	2.32
Q07866	KLC1	0.94	3.86
O60563	CCNT1	0.93	4.77
Q96LB3	IFT74	0.93	3.83
P49756	RBM25	0.92	3.43
Q9UQR1	ZNF148	0.92	2.89
P49790	NUP153	0.91	2.50
Q7LBC6	KDM3B	0.91	4.78
Q9NW82	WDR70	0.91	3.53
P35269	GTF2F1	0.91	4.59
Q96QC0	PPP1R10	0.90	5.96
Q9Y5Q8	GTF3C5	0.89	4.72
Q92769	HDAC2	0.88	4.78
P28290	ITPRID2	0.88	5.51
Q6UXN9	WDR82	0.88	4.66
Q12979	ABR	0.87	4.81
P98175	RBM10	0.87	4.87
Q9UJX2	CDC23	0.87	3.77
Q15459	SF3A1	0.85	3.65
Q86X02	CDR2L	0.85	4.67
A0A060ADZ4	RSV P	0.85	4.43
P43243	MATR3	0.83	3.65
O15067	PFAS	0.82	4.16
Q9HCK8	CHD8	0.82	4.59
Q8IWX8	CHERP	0.82	5.51
Q13416	ORC2	0.81	4.00
Q01780	EXOSC10	0.80	5.06

For Supplementary Table S2 and S3 we refer to the separate excel files. Supplementary Table S2 and S3 respectively show the results of the primary MAPPIT and KISS screen against the 15k ORF prey collection.

Both Suppl. Table S2 and S3 consist of 3 excel tabs: 'AllPlatesWithoutControlNormaliz', 'hits_Q_below0.35' and 'filtered_list'. The first tab (AllPlatesWithoutControlNormaliz) shows the results of the raw data of the screen against the complete ORF library. The second tab (hits_Q_below0.35) shows a subset of tab 1 where only preys are retained that give a Q value below 0.35. The third tab (filtered_list) shows a subset of tab 2, where only preys are retained with particle counts higher than two and where known aspecific binders are removed.

In the ORF prey collection, several empty preys are present. These preys have not been sequenced and are therefore considered as empty. When we upload the filtered datasets (q value <0.35, particle counts > 2, removal of known aspecific binders) of the MAPPIT and KISS primary screen into the jvenn online tool to identify common proteins between the performed screens, all empty preys are considered as one prey since no distinction is made by the tool. This accounts for the small differences in protein numbers that are listed in this filtered list (388 preys in Suppl. Table S2 (MAPPIT); 484 preys in Suppl. Table S3 (KISS)) and the protein numbers depicted by the Venn diagram (Fig.4) (380 preys for MAPPIT; 475 preys for KISS).

Since none of the empty preys was identified by both MAPPIT and KISS, we did not further investigate the possible identity of the preys.

Supplementary Table S4. Custom generated prey list for MAPPIT and KISS retests. This pick list includes interesting candidates identified in the BioID screen as well as published NS1 interactors. Also several key proteins (possibly) involved in the induction and signaling of type I and type III IFN upon RSV infection were included in this list. The preys highlighted in green correspond to the NS1-specific interactions as shown in Fig. 3C (MAPPIT) and 3D (KISS).

	MAPPIT							KISS						
Entrez_ID	VALUE PIB	VALUE BP	BP/PIB	BP/BIP	Lowest value (MIN BP/PIB and BP/BIP)	PIB/IBIP	Score	VALUE PIB	VALUE BP	BP/PIB	BP/BIP	Lowest value (MIN BP/PIB and BP/BIP)	PIB/IBIP	Score
SEC31A	1.10	1.71	1.55	1.61	1.55	1.20	-	11960.00	6259.75	0.52	0.75	0.52	1.17	-
STAT1	0.94	1.17	1.24	1.10	1.10	1.03	-	3026.75	2283.25	0.75	0.27	0.27	0.30	-
cullin-2	1.89	10.42	5.50	9.78	5.50	2.06	-	10186.50	4940.00	0.48	0.59	0.48	1.00	-
TBK1	1.01	1.23	1.23	1.16	1.16	1.10	-	8613.25	5506.75	0.64	0.66	0.64	0.84	-
GR	1.02	1.05	1.03	0.98	0.98	1.11	-	3560.25	3313.25	0.93	0.40	0.40	0.35	-
OSBPL8	1.00	1.65	1.66	1.55	1.55	1.08	-	8816.50	6076.50	0.69	0.73	0.69	0.86	-
TLR3	1.00	1.14	1.14	1.07	1.07	1.08	-	4050.25	3783.50	0.93	0.45	0.45	0.40	-
PPP1R12A	1.02	1.37	1.34	1.28	1.28	1.11	-	4233.25	3590.00	0.85	0.43	0.43	0.41	-
MDA-5	1.15	3.87	3.36	3.63	3.36	1.25	-	3666.50	5443.25	1.48	0.65	0.65	0.36	-
H2BD	0.96	1.21	1.27	1.14	1.14	1.04	-	3776.75	2626.50	0.70	0.31	0.31	0.37	-
PFDN2	24.38	67.82	2.78	63.67	2.78	26.53	A	5596.50	5083.25	0.91	0.61	0.61	0.55	-
FTL	1.00	5.79	5.82	5.44	5.44	1.08	-	5853.25	4733.50	0.81	0.57	0.57	0.57	-
RAC1	1.76	8.51	4.83	7.99	4.83	1.92	-	8530.00	12586.75	1.48	1.51	1.48	0.83	-
SNAP23	1.02	1.12	1.10	1.06	1.06	1.11	-	3500.00	3906.50	1.12	0.47	0.47	0.34	-
MSRA	1.62	97.05	59.97	91.11	59.97	1.76	+	9280.00	86246.75	9.29	10.34	9.29	0.91	+
CPSF4	1.03	4.72	4.58	4.43	4.43	1.12	-	16876.75	18973.50	1.12	2.27	1.12	1.65	-
C17orf82	1.59	15.42	9.70	14.47	9.70	1.73	+	8773.50	11346.50	1.29	1.36	1.29	0.86	-
AZU1	1.02	99.91	97.58	93.79	93.79	1.11	+	4746.75	23910.00	5.04	2.87	2.87	0.46	-
PSME3	0.98	1.18	1.20	1.11	1.11	1.06	-	3196.75	3436.75	1.08	0.41	0.41	0.31	-
RNF144A	16.12	58.26	3.61	54.70	3.61	17.54	A	5810.00	4763.25	0.82	0.57	0.57	0.57	-
PSMB4	1.04	2.24	2.16	2.11	2.11	1.13	-	7653.25	15260.25	1.99	1.83	1.83	0.75	-
PAPOLA	1.06	1.21	1.14	1.14	1.14	1.15	-	7093.25	7690.00	1.08	0.92	0.92	0.69	-
MyD88	1.12	1.39	1.24	1.30	1.24	1.22	-	7026.75	5950.00	0.85	0.71	0.71	0.69	-
RRAGA	2.91	33.17	11.41	31.14	11.41	3.16	+	8113.50	11837.00	1.46	1.42	1.42	0.79	-
ZFC3H1	1.01	1.32	1.31	1.24	1.24	1.10	-	1853.25	1503.25	0.81	0.18	0.18	0.18	-
TRIM16L	1.07	3.09	2.90	2.90	2.90	1.16	-	7210.00	5663.25	0.79	0.68	0.68	0.70	-
HP1BP3	0.96	1.19	1.24	1.12	1.12	1.04	-	3526.50	3173.25	0.90	0.38	0.38	0.34	-
PFDN5	0.99	7.06	7.13	6.63	6.63	1.08	-	6303.25	3783.25	0.60	0.45	0.45	0.62	-
WDR33	0.89	1.38	1.54	1.30	1.30	0.97	-	2776.50	1930.00	0.70	0.23	0.23	0.27	-
PCGF2	1.33	3.94	2.97	3.69	2.97	1.44	-	10503.25	8343.50	0.79	1.00	0.79	1.03	-
TMOD3	0.93	1.14	1.24	1.07	1.07	1.01	-	3496.75	3520.00	1.01	0.42	0.42	0.34	-
SURF4	0.87	1.10	1.27	1.03	1.03	0.94	-	3006.75	3336.50	1.11	0.40	0.40	0.29	-
GTF2E2	1.11	1.06	0.96	1.00	0.96	1.21	-	770.00	1230.00	1.60	0.15	0.15	0.08	-
CCNC	1.02	1.13	1.11	1.06	1.06	1.11	-	1983.25	2550.00	1.29	0.31	0.31	0.19	-
ANXA4	2.39	15.68	6.55	14.72	6.55	2.60	-	9627.00	9940.00	1.03	1.19	1.03	0.94	-
PRPS2	1.28	13.22	10.32	12.41	10.32	1.39	+	4803.50	7286.50	1.52	0.87	0.87	0.47	-
NDNF	1.32	1.85	1.40	1.73	1.40	1.44	-	3456.75	5166.50	1.49	0.62	0.62	0.34	-
PSMD4	1.06	1.00	0.94	0.94	0.94	1.15	-	3286.50	3793.25	1.15	0.45	0.45	0.32	-
RBFOX2	5.62	37.74	6.71	35.43	6.71	6.12	-	24530.25	21273.25	0.87	2.55	0.87	2.40	-
RASSF8	0.90	1.10	1.22	1.03	1.03	0.98	-	11683.50	14893.50	1.27	1.79	1.27	1.14	-
TRIF	1.04	1.01	0.98	0.95	0.95	1.13	-	1190.00	1470.00	1.24	0.18	0.18	0.12	-
FBXO9	2.29	10.83	4.73	10.17	4.73	2.49	-	5593.50	8363.50	1.50	1.00	1.00	0.55	-
PTPRK	4.34	42.87	9.88	40.25	9.88	4.72	+	9227.00	9376.75	1.02	1.12	1.02	0.90	-
STAT5B	1.88	14.93	7.96	14.02	7.96	2.04	-	7616.75	14610.00	1.92	1.75	1.75	0.74	-
PLEKHO2	1.39	11.99	8.62	11.26	8.62	1.51	-	12169.75	13880.00	1.14	1.66	1.14	1.19	-
PDK1	1.14	4.60	4.05	4.32	4.05	1.24	-	11013.50	16933.25	1.54	2.03	1.54	1.08	-

SNX4	2.38	24.42	10.27	22.93	10.27	2.59	+	15843.50	17910.00	1.13	2.15	1.13	1.55	-
IRF3	1.08	3.15	2.90	2.95	2.90	1.18	-	29333.25	28593.50	0.97	3.43	0.97	2.87	-
GOPC	1.06	1.06	1.00	1.00	1.00	1.16	-	1996.75	2060.00	1.03	0.25	0.25	0.20	-
CPSF7	0.98	1.03	1.05	0.97	0.97	1.07	-	2846.75	6083.00	2.14	0.73	0.73	0.28	-
CPSF6	1.00	1.02	1.02	0.96	0.96	1.09	-	1520.25	2340.00	1.54	0.28	0.28	0.15	-
ZBTB9	1.05	1.28	1.23	1.21	1.21	1.14	-	11593.25	23949.75	2.07	2.87	2.07	1.13	-
DNAJC7	0.87	1.03	1.18	0.97	0.97	0.95	-	13340.00	17083.25	1.28	2.05	1.28	1.30	-
CELF5	3.18	4.85	1.53	4.55	1.53	3.46	-	37616.75	22403.25	0.60	2.69	0.60	3.68	-
OSBPL6	3.30	19.63	5.96	18.42	5.96	3.59	-	13170.00	15486.75	1.18	1.86	1.18	1.29	-
FARSB	1.26	11.01	8.76	10.34	8.76	1.37	-	6953.25	7486.75	1.08	0.90	0.90	0.68	-
IRF9	0.97	0.98	1.01	0.92	0.92	1.05	-	1790.00	2140.00	1.20	0.26	0.26	0.17	-
ACTR3	0.98	2.37	2.41	2.22	2.22	1.07	-	6370.00	7806.50	1.23	0.94	0.94	0.62	-
CCT5	0.92	1.39	1.51	1.30	1.30	1.00	-	2343.50	3556.50	1.52	0.43	0.43	0.23	-
CCT6A	6.04	20.03	3.32	18.80	3.32	6.57	-	4770.00	7910.00	1.66	0.95	0.95	0.47	-
STIP1	0.78	1.14	1.46	1.07	1.07	0.85	-	2429.75	3776.75	1.55	0.45	0.45	0.24	-
POLR2A	2.46	10.15	4.13	9.53	4.13	2.68	-	7400.00	14903.25	2.01	1.79	1.79	0.72	-
TOM1	0.86	1.13	1.32	1.07	1.07	0.93	-	2640.00	3663.25	1.39	0.44	0.44	0.26	-
RBM6	0.88	1.38	1.57	1.30	1.30	0.95	-	5050.25	15739.75	3.12	1.89	1.89	0.49	-
FRMD6	20.49	130.36	6.36	122.38	6.36	22.29	A	2623.25	5450.25	2.08	0.65	0.65	0.26	-
CSTF2T	1.21	6.23	5.13	5.85	5.13	1.32	-	7143.25	9336.75	1.31	1.12	1.12	0.70	-
IKKe	0.90	2.18	2.42	2.04	2.04	0.98	-	5049.75	5153.50	1.02	0.62	0.62	0.49	-
TBRG4	2.18	22.25	10.20	20.88	10.20	2.37	+	4390.00	7880.00	1.79	0.94	0.94	0.43	-
HSPA2	1.00	8.35	8.31	7.84	7.84	1.09	-	2990.00	6126.75	2.05	0.73	0.73	0.29	-
CEP72	0.82	1.75	2.14	1.64	1.64	0.89	-	64260.00	75460.00	1.17	9.04	1.17	6.28	A
VILL	3.61	51.29	14.22	48.16	14.22	3.92	+	8650.25	15669.75	1.81	1.88	1.81	0.85	-
AMOT	0.89	1.65	1.85	1.55	1.55	0.97	-	50683.25	41406.75	0.82	4.96	0.82	4.95	-
DTX3L	14.59	119.88	8.21	112.54	8.21	15.88	A	5773.50	13087.00	2.27	1.57	1.57	0.56	-
ADD3	4.92	36.99	7.52	34.73	7.52	5.35	-	5620.25	8596.75	1.53	1.03	1.03	0.55	-
STAT2	1.88	7.75	4.12	7.28	4.12	2.05	-	4476.75	7046.75	1.57	0.84	0.84	0.44	-
EIF4E3	1.07	6.23	5.80	5.85	5.80	1.17	-	7490.25	12096.75	1.61	1.45	1.45	0.73	-
COMMD4	3.53	22.15	6.27	20.79	6.27	3.84	-	13926.75	16583.50	1.19	1.99	1.19	1.36	-
COMMD5	1.73	10.48	6.04	9.84	6.04	1.89	-	15003.50	18706.75	1.25	2.24	1.25	1.47	-
IQCB1	2.00	9.28	4.63	8.71	4.63	2.18	-	11556.50	20753.25	1.80	2.49	1.80	1.13	-
CPSF2	0.95	1.27	1.34	1.19	1.19	1.03	-	3003.50	4376.50	1.46	0.52	0.52	0.29	-
NSFL1C	1.05	1.20	1.15	1.12	1.12	1.14	-	926.75	1673.50	1.81	0.20	0.20	0.09	-
NOL4	0.98	38.35	38.98	36.00	36.00	1.07	+	4230.00	96760.00	22.87	11.60	11.60	0.41	+
RBFOX1	0.97	1.25	1.29	1.18	1.18	1.06	-	39120.00	28130.00	0.72	3.37	0.72	3.82	-
ZBTB44	23.88	110.99	4.65	104.20	4.65	25.98	A	3596.50	7190.00	2.00	0.86	0.86	0.35	-
TRIM67	0.99	1.69	1.71	1.58	1.58	1.08	-	21296.75	29396.50	1.38	3.52	1.38	2.08	-
ZMYM2	0.92	1.16	1.26	1.09	1.09	1.00	-	3867.00	5013.50	1.30	0.60	0.60	0.38	-
MED25	1.32	14.50	10.95	13.62	10.95	1.44	+	7013.25	94756.75	13.51	11.36	11.36	0.69	+
GMEB2	0.98	1.18	1.20	1.11	1.11	1.07	-	3116.50	5740.00	1.84	0.69	0.69	0.30	-
SSX4	1.51	10.08	6.69	9.46	6.69	1.64	-	7096.50	17620.00	2.48	2.11	2.11	0.69	-
NOD2	2.51	5.75	2.29	5.40	2.29	2.73	-	3443.50	3096.75	0.90	0.37	0.37	0.34	-
DOCK7	0.96	1.08	1.12	1.02	1.02	1.05	-	2133.25	2346.75	1.10	0.28	0.28	0.21	-
EP300	6.42	58.16	9.05	54.60	9.05	6.99	+	12796.75	16210.00	1.27	1.94	1.27	1.25	-
map1b	0.93	1.15	1.23	1.08	1.08	1.01	-	1793.25	2903.25	1.62	0.35	0.35	0.18	-
hRSV-NS1	1.35	2.65	1.96	2.49	1.96	1.47	-	1686.75	2946.75	1.75	0.35	0.35	0.16	-
hRSV-NS2	2.05	12.60	6.16	11.83	6.16	2.23	-	11860.00	12176.75	1.03	1.46	1.03	1.16	-
ARP2	1.54	10.40	6.75	9.76	6.75	1.67	-	9290.00	8100.00	0.87	0.97	0.87	0.91	-

Supplementary Table S5. Candidate interactors of NS1 identified in at least 2 or all 3 of the 3 protein-protein mapping techniques (*i.e.* the BioID -, MAPPIT – and KISS screen) with a description of their function.

Identified in BioID, MAPPIT and KISS

Protein ID	Gene name	Function
P33215	NEDD1	Required for mitosis progression. Promotes the nucleation of microtubules from the spindle.
Q71SY5	MED25	Component of the Mediator complex, a coactivator involved in the regulated transcription of nearly all RNA polymerase II-dependent genes.
P09913	IFIT2	IFN-induced antiviral protein which inhibits expression of viral messenger RNAs lacking 2'-O-methylation of the 5' cap.

Identified in MAPPIT and KISS

Protein ID	Gene name	Function
Q14331	FRG1B	Binds to mRNA in a sequence-independent manner.
Q9UJ68	MSRA	Has an important function as a repair enzyme for proteins that have been inactivated by oxidation.
P08236	GUSBP2	Plays an important role in the degradation of dermatan and keratan sulfates.
P20160	AZU1	This is a neutrophil granule-derived antibacterial and monocyte- and fibroblast-specific chemotactic glycoprotein.
Q6DKK2	TTC19	Required for the preservation of the structural and functional integrity of mitochondrial respiratory complex III.
P51692	STAT5B	Carries out a dual function: signal transduction and activation of transcription.
P63000	RAC1	Plasma membrane-associated small GTPase which cycles between active GTP-bound and inactive GDP-bound states.
Q709F0	ACAD11	Acyl-CoA dehydrogenase, that exhibits maximal activity towards saturated C22-CoA.
O94818	NOL4	Predicted to enable RNA binding activity.
Q9UEY8	ADD3	Membrane-cytoskeleton-associated protein that promotes the assembly of the spectrin-actin network.
P11908	PRPS2	Catalyzes the synthesis of phosphoribosylpyrophosphate (PRPP) that is essential for nucleotide synthesis.
Q8N0Z2	ABRA	Acts as an activator of serum response factor (SRF)-dependent transcription through a mechanism requiring Rho-actin signaling.
Q8TDN6	BRIX1	Required for biogenesis of the 60S ribosomal subunit.
Q8NCF0	CLEC18C	Binds polysaccharidesin in a Ca^{2+}-independent manner.
Q9GZQ3	COMMD5	May modulate activity of cullin-RING E3 ubiquitin ligase (CRL) complexes.
Q5FBB7	SGOL1	Plays a central role in chromosome cohesion during mitosis.
Q5T9G4	ARMC12	Required for proper mitochondrial elongation and coiling along the flagellum during the formation of the mitochondrial sheath.
P17252	PRKCA	Involved in cell proliferation and cell growth arrest by positive and negative regulation of the cell cycle.
Q9HAI6	CXorf21	Innate immune adapter that mediates the recruitment and activation of IRF5 downstream of endolysosomal toll-like receptors TLR7, TLR8 and TLR9.
Q96QD5	DEPDC7	Predicted to be involved in intracellular signal transduction.
Q96MC5	C16orf45	Involved in negative regulation of microtubule depolymerization.

Q86Z20	CCDC125	May be involved in the regulation of cell migration
P08238	HSP90AB1	Molecular chaperone that promotes the maturation, structural maintenance and proper regulation of specific target proteins involved for instance in cell cycle control and signal transduction.
Q9UI47	CTNNA3	May be involved in formation of stretch-resistant cell-cell adhesion complexes.
Q9P0J7	KCMF1	Has intrinsic E3 ubiquitin ligase activity and promotes ubiquitination.
Q8IXS2	CCDC65	Component of the nexin-dynein regulatory complex (N-DRC), a key regulator of ciliary/flagellar motility.
P36897	TGFBR1	Transmembrane serine/threonine kinase forming with the TGF-beta type II serine/threonine kinase receptor.
Q6IPU0	CENPP	Component of the CENPA-CAD (nucleosome distal) complex, a complex recruited to centromeres which is involved in assembly of kinetochore proteins, mitotic progression and chromosome segregation.
Q5T5P2	KIAA1217	Required for normal development of intervertebral disks.
Q9NR12	PDLIM7	May function as a scaffold on which the coordinated assembly of proteins can occur.
Q9H4G0	EPB41L1	Involved in actin binding and actomyosin structure organization.
Q9UQM7	CAMK2A	Calcium/calmodulin-dependent protein kinase that functions autonomously after Ca2+/calmodulin-binding and autophosphorylation.
P40937	RFC5	Smallest subunit of the replication factor C complex, which is required for DNA replication.
Q5VZQ5	C10orf122	Testis-Expressed Protein 36
Q5MJ09	SPANXN3	Belongs to the SPAN-X family.
Q86TB9	PATL1	RNA-binding protein involved in deadenylation-dependent decapping of mRNAs, leading to the degradation of mRNAs.
P61201	COPS2	Essential component of the COP9 signalosome complex (CSN), an essential regulator of the ubiquitin (Ubl) conjugation pathway.
Q1A5X7	WHAMMP3	Predicted to enable Arp2/3 complex binding activity.
Q8TEC5	SH3RF2	Has E3 ubiquitin-protein ligase activity.
Q96ST8	CEP89	Required for ciliogenesis.
P01350	GAST	Gastrin stimulates the stomach mucosa to produce and secrete hydrochloric acid and the pancreas to secrete its digestive enzymes.
Q5M9N0	CCDC158	Coiled-coil domain-containing protein 158.
Q96JC1	VPS39	Plays a role in vesicle-mediated protein trafficking to lysosomal compartments including the endocytic membrane transport and autophagic pathways.
Q86W26	NLRP10	Involved in the innate immune response by contributing to proinflammatory cytokine release in response to invasive bacterial infection.
Q96BM1	ANKRD9	Substrate receptor subunit of a cullin-RING superfamily E3 ligase complex (CUL5-based E3 ubiquitin ligase complex).
Q96PU4	UHRF2	E3 ubiquitin ligase that plays important roles in DNA methylation, histone modifications, cell cycle and DNA repair.
(?)	MORC2-AS1	Affiliated with the lncRNA class.
P01834	IGKC	Constant region of immunoglobulin light chains.
P42357	HAL	Involved in the histidine catabolic process.
Q96M63	CCDC114	Component of the outer dynein arm-docking complex (ODA-DC).
Q8NCS7	SLC44A5	Involved in choline transmembrane transporter activity.
Q9NSU2	TREX1	Major cellular 3'-to-5' DNA exonuclease which digests single-stranded DNA (ssDNA) and double-stranded DNA (dsDNA) with mismatched 3' termini.
Q9H0B3	KIAA1683	IQ motif containing N. Function unknown.

Q9Y6H3	XRCC6BP1	Involved in double-strand break repair via nonhomologous end joining.
Q86XK2	FBXO11	Substrate recognition component of a SCF (SKP1-CUL1-F-box protein) E3 ubiquitin-protein ligase complex.
O95210	STBD1	Acts as a cargo receptor for glycogen.
Q8NA92	THAP8	Predicted to enable DNA binding activity and metal ion binding activity.
Q8NCA5	FAM98A	Involved in protein methylation and positive regulation of cell population proliferation.
(?)	LOC254896	Affiliated with the lncRNA class.
P28715	ERCC5	Single-stranded structure-specific DNA endonuclease involved in DNA excision repair.
P48668	KRT6C	Involved in intermediate filament cytoskeleton organization.
Q9Y2Y8	PRG3	Involved in several processes, including granulocyte activation; histamine biosynthetic process; and regulation of gene expression.
P29084	GTF2E2	Recruits TFIIH to the initiation complex and stimulates the RNA polymerase II C-terminal domain kinase and DNA-dependent ATPase activities of TFIIH.
Q8IWU5	SULF2	Exhibits arylsulfatase activity and highly specific endoglucosamine-6-sulfatase activity.
P42127	ASIP	Involved in the regulation of melanogenesis.

**Identified in BioID and MAPPIT**		
**Protein ID**	**Gene name**	**Function**
Q9H9A5	CNOT10	Component of the CCR4-NOT complex which is one of the major cellular mRNA deadenylases.
Q8TDB6	DTX3L	E3 ubiquitin-protein ligase which, in association with ADP-ribosyltransferase PARP9, plays a role in DNA damage repair and in interferon-mediated antiviral responses.
Q9H0L4	CSTF2T	Involved in pre-mRNA cleavage required for polyadenylation.

**Identified in BioID and KISS**		
**Protein ID**	**Gene name**	**Function**
Q07866	KLC1	Has cytoskeletal motor activity.
Q02241	KIF23	Essential for cytokinesis in Rho-mediated signaling.
P86790	CCZ1B	Involved in vesicle-mediated transport.
Q8N511	TMEM199	Accessory component of the proton-transporting vacuolar (V)-ATPase protein pump involved in intracellular iron homeostasis.
Q96JH7	VCPIP1	Deubiquitinating enzyme involved in DNA repair and reassembly of the Golgi apparatus and the endoplasmic reticulum following mitosis.
Q13501	SQSTM1	May be involved in cell differentiation, apoptosis, immune response and regulation of K+ channels.

References

1. Lievens S, Heyden V Der, Masschaele D, Ceuninck L De, Petta I, Gupta S, et al. Proteome-scale Binary Interactomics in Human Cells. Mol Cell Proteomics. 2016;15(12):3624–39.
